# 1,2-Benzenedithiol and Toluene-3,4-dithiol Arsenic(III) Complexes—Synthesis, Structure, Spectroscopic Characterization and Toxicological Studies

**DOI:** 10.3390/molecules24213865

**Published:** 2019-10-26

**Authors:** Monika Lyczko, Krzysztof Lyczko, Agnieszka Majkowska-Pilip, Aleksander Bilewicz

**Affiliations:** Institute of Nuclear Chemistry and Technology, Dorodna 16, 03-195 Warsaw, Poland; k.lyczko@ichtj.waw.pl (K.L.); a.majkowska@ichtj.waw.pl (A.M.-P.); a.bilewicz@ichtj.waw.pl (A.B.)

**Keywords:** arsenic(III) complexes, dithiols, crystal structure, DFT calculations, cytotoxicity, APL, NB4 cells

## Abstract

A new group of arsenic(III) complexes with bidentate S,S-donor ligands, 1,2-benzenedithiol (Ph(SH)_2_) and toluene-3,4-dithiol (MePh(SH)_2_), were synthesized. The use of arsenic(III) iodide and bromide promoted the formation of neutral complexes (**1**–**4**) with the general formula AsX(LS_2_) (X = I or Br, L = MePh or Ph). The crystal structures of these compounds were determined using single-crystal X-ray diffraction (scXRD). Unlike other arsenic(III) complexes, AsBr(PhS_2_) complex (**2**) was found to crystallize with a rare 13 molecules in the asymmetric unit. The compounds were also characterized by conventional physico-chemical techniques (Fourier transform infrared (FT-IR) spectroscopy, ultraviolet-visible (UV-Vis) spectroscopy, nuclear magnetic resonance (NMR), high-performance liquid chromatography (HPLC), elemental analysis (EA) and electrospray ionization-mass spectrometry (ESI-MS)). The results from structural and spectroscopic studies were supported by DFT calculations using the B3LYP/LANL2DZ and (or) 6-31+G(d,p) approaches. The cytotoxicity of these complexes was estimated for human acute promyelocytic leukemia cell line (NB4). They exhibited remarkable cytotoxicities after 48 h of treatment with IC_50_ equal to about 10 µM and 40 µM for complexes with 1,2-benzenedithiolato and toluene-3,4-dithiolato ligand, respectively. Their toxicity was lower than that of commonly used chemotherapeutic As_2_O_3_ (IC_50_ = 1.4 µM).

## 1. Introduction

Inorganic arsenic exhibits strong toxic properties and is classified by the International Agency for Research on Cancer and US Environmental Protection Agency as a human carcinogen. Arsenic causes huge cellular alterations via numerous pathways, such as induction of apoptosis, inhibition of proliferation, stimulation of differentiation, and inhibition of angiogenesis [1]. Although arsenic is highly toxic for humans, for years it has been employed in medicine to treat many diseases. The affinity of arsenic to sulphur atoms is of great importance for the development of new arsenic compounds, which are applicable in cancer therapy. Furthermore, the search for novel compounds possessing lower toxicity for patients compared with classic As_2_O_3_, which is currently used for the treatment of acute promyelocytic leukemia (APL) as well as other types of cancers, is desirable [2]. However, chronic exposure to arsenic or arsenic derivatives can also promote toxic effects, because similar mechanisms are responsible for both the therapeutic and toxic effects.

The As_2_O_3_ mechanism of action is complicated and leads to induction of apoptosis and partial differentiation of cells. The binding of trivalent arsenic to crucial thiol groups in proteins may inhibit biochemical events and cause toxicity. On the other hand, binding arsenite at non-essential sites may involve a detoxication mechanism. Reports have shown that an acute toxicity of arsenic is related to its chemical form and oxidation state. Inorganic arsenic in its oxidation state +5 is metabolized by the process of a two-electron reduction to arsenic(III), and then by an oxidative methylation to pentavalent organic arsenic [3].

Trivalent arsenicals readily react with thiol-containing molecules. Unfortunately, specific functional groups within enzymes, receptors or coenzymes, such as thiols or vicinal sulfhydryls, have a major role in the activity of these molecules which results in the attachment of arsenic(III) causing reduction in their biological activity [3,4].

Glutathione (GSH) plays an important role in the metabolism of arsenic in human tissues by forming stable complexes with arsenic. The sensitivity of cells toward arsenic compounds is related to the level of GSH in the cell [5,6]. Therefore, cancer cells that contain low levels of GSH are arsenic sensitive, in contrast, those with higher GSH concentration, such as lung and liver cancer cells, are more arsenic resistant [7]. Therefore, GSH in human cells inhibit arsenic(III) toxicity.

There are many arsenic(III) complexes possessing organic compounds, which involve two S donor atoms, such as dithiocarbamates, where arsenic is bound bidently or monodently [8,9,10,11,12,13,14]. The bidentate sulphur donor ligands can form four- [8,14,15], five- [16,17,18], six- [17,18] and even seven-membered [17,19] chelate rings with arsenic(III) ion. Furthermore, although scarce, homoleptic arsenic(III) compounds with monodently acting S donor ligands have been structurally characterized [17,20,21,22,23].

Recently, the synthesis and structure of chloro-arsenic(III) compounds with toluene-3,4-dithiolato and 1,2-benzenedithiolato ligands have been published [24]. However, Kisenyi et al. were the first to report this compound over 30 years ago [25]. Besides these chloro-arsenic(III) species the simple salts of [As(MePhS_2_)][MCl_4_] (M = Al, Ga) and [As(PhS_2_)_2_][NEt_4_] have also been presented [24,26].

In the case of toluene-3,4-dithiol some dithiocarbamate and dithiophosphate derivatives of arsenic(III) compounds [As(S_2_CNR_2_)(MePhS_2_) and As(S_2_P(OmTol)_2_)(MePhS_2_)] are also structurally characterized [13,27].

Therefore, arsenic complexes with dithiols as well as As_2_O_3_ could be an interesting cytostatic agents for tumor treatment. Since dithiols form complexes with As(III) via two sulphur atoms, the last site could be occupied by any anion or used as a convenient linker with the suitable biomolecule. This would allow the conjugation of an appropriate compound that could, for example, be a guiding molecule to specific cancer cells. This type of complexation would be also helpful for their application in targeted radionuclide therapy. Recently, due to the development of personalized medicine radioisotopes of arsenic, they are of considerable interest to the field of nuclear medicine with unique nuclear properties making them well-suited for novel theranostic radiopharmaceuticals. The decay properties of arsenic radionuclides are ideal for a variety of diagnostic and therapeutic applications. Radionuclides ^77^As (β^–^ emitter with T_1/2_ = 38.83 h and energy E_βmax_ = 0.683 MeV) and ^72^As (positron emitter with T_1/2_ = 26.0 h and energy E_γ_ = 511, 834 keV) form an ideal theranostic pair. These radionuclides, when attached to an appropriate biomolecule such as a monoclonal antibody, can be used in cancer diagnosis, plan treatment, and provide effective therapy [28]. In this work we present arsenic(III) complexes with selected dithiols for possible therapeutic applications. After full structural characterization, the cytotoxicity of the obtained complexes for acute promyelocytic leukemia cells (NB4) was investigated and compared with As_2_O_3_.

## 2. Results and Discussion

### 2.1. Synthesis

**Scheme 1:**
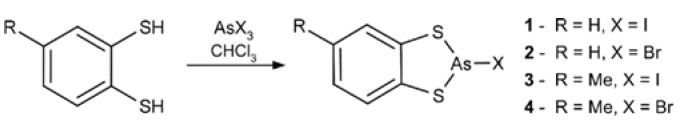
Synthesis of dithiolato arsenic(III) compounds.

**Figure 1:**
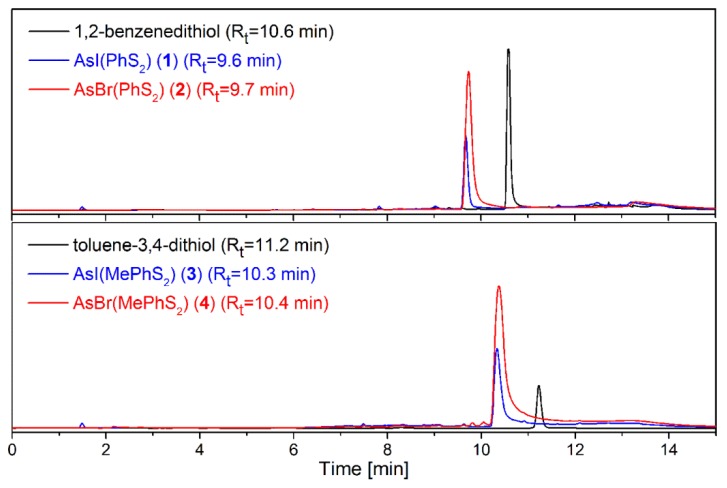
The high-performance liquid chromatography (HPLC) chromatograms of the obtained arsenic compounds.

Four new arsenic(III) complexes were obtained as orange (**1** and **3**) or yellow (**2** and **4**) crystals in moderate yields (40–57%) via the reaction of two different dithiol ligands (1,2-benzenedithiol (Ph(SH)_2_) and toluene-3,4-dithiol (MePh(SH)_2_)) and arsenic(III) compounds (AsBr_3_ and AsI_3_) in refluxing chloroform solutions. Scheme 1 shows a simplified reaction pathway of the prepared compounds. The synthesis of these compounds was inspired by the methods described in ref [25]. All complexes crystalized quickly, typically overnight and their structures confirmed using common methods (single-crystal X-ray diffraction (scXRD), elemental analysis (EA), electrospray ionization-mass spectrometry (ESI-MS), Fourier transform infrared spectroscopy (FT-IR), ultraviolet-visible (UV-Vis) spectroscopy, nuclear magnetic resonance (NMR)). High-performance liquid chromatography (HPLC) reference peaks were also determined for every compound (Figure 1). The retention times of 1–4 differ from the free dithiols, so the reaction progress is easy to control via this simple method.

### 2.2. Description of the Prepared Complexes Molecular and Crystal Structures

**Figure 2:**
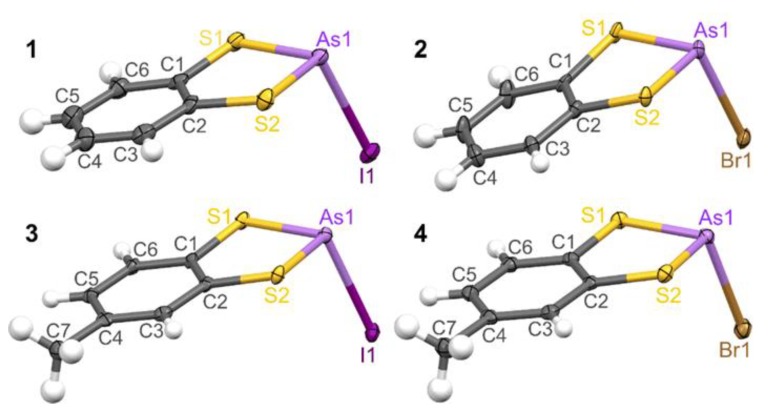
Molecular structure of compounds **1**–**4**.

The main bond lengths and angles derived from measurements and calculations of compounds **1**–**4** are shown in Table 1, whereas the molecular structure of these compounds are presented in Figure 2. In each individual molecule of the studied complexes the arsenic atom is three coordinate, being bound to a halogen atom and chelated to two sulphur atoms belonging to the dithiolato ligand. The arsenic(III) coordination sphere can therefore be described as distorted tetrahedral, with one corner occupied by the active 4*s*^2^ lone electron pair. The mean As–S bond length (2.21 Å) in **1**–**4** is about 0.26 and 0.50 Å shorter than the mean As–Br (2.47 Å) and As–I (2.71 Å) distances, respectively. All determined bond lengths are similar to the corresponding distances in other arsenic(III) compounds [16,17,29]. Three angles with the apex on the arsenic atom changing in range 93°–106° are less than the normal tetrahedral angle (109.5°) showing the dominant position of the lone electron pair on the central atom. The S1–As1–S2 bite angles (mean value of 93.2°) are typical of this type of five-membered chelate ring [17,24]. All studied compounds have a non-planar chelate ring (envelope geometry) with the arsenic atom lying approximately 0.22, 0.31 (mean value from 13 molecules), 0.32 and 0.33 Å out of plane formed by the S1, C1, C2 and S2 atoms for **1**–**4**, respectively.

**Figure 3:**
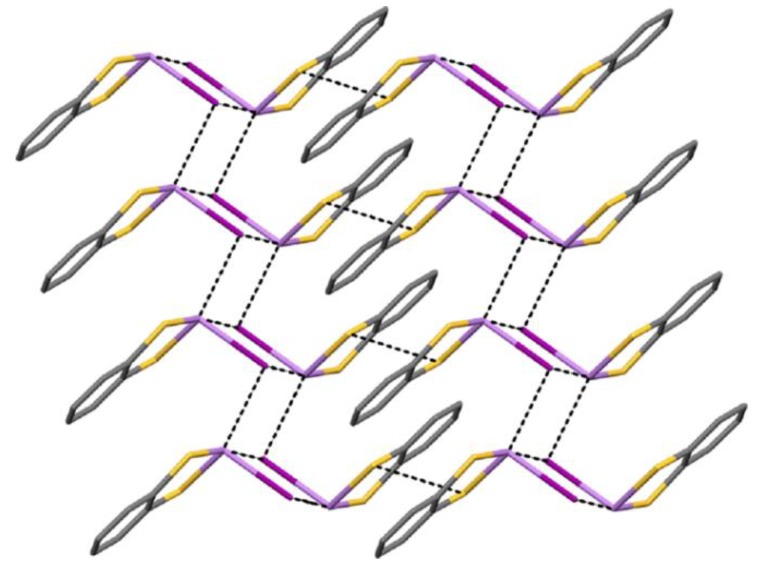
Fragment of the crystal structure of **1** showing some intermolecular interactions between AsI(PhS_2_) molecules.

**Figure 4:**
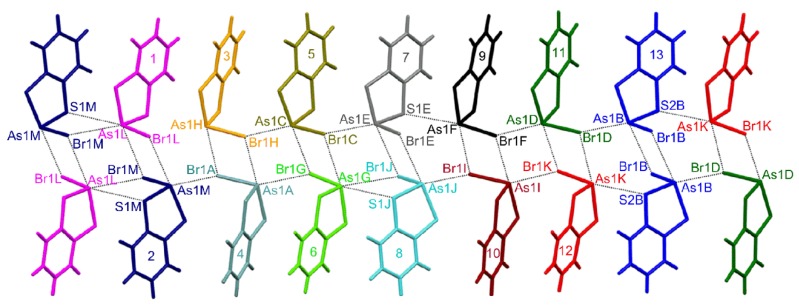
Fragment of a chain in compound **2** showing As···Br and As···S close contacts. Molecules are presented in different colors to highlight the 13 crystalographically independent molecules in the asymmetric unit.

**Figure 5:**
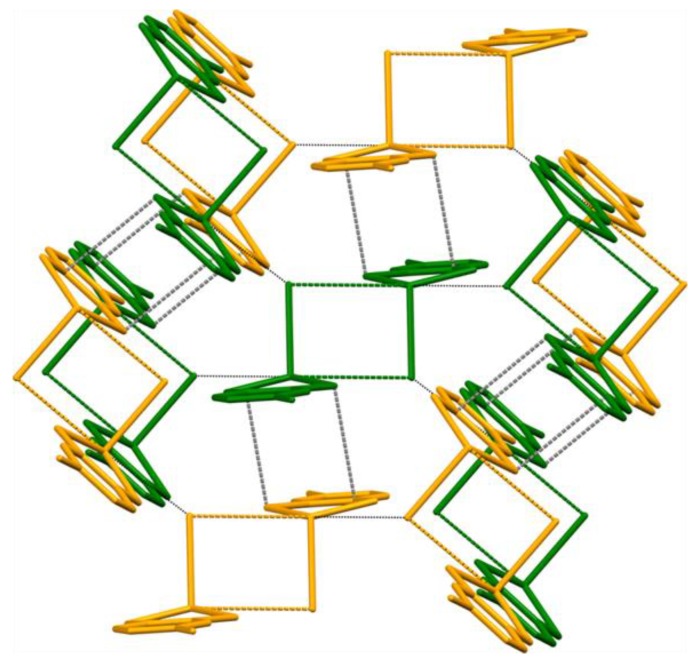
Fragment of the crystal structure of **3** and **4** showing some intermolecular interactions.

The main motif in all presented crystal structures is a dimeric fragment [AsX(LS_2_)]_2_ (X = I or Br, L = MePh or Ph). Within these dimeric units the molecules are stabilized through four-center [AsX]_2_ interactions. Compound **1** has a crystal structure consisting of chains along the crystallographic axis formed by the repetition of one [AsI(PhS_2_)]_2_ fragment (Figure 3) which is stabilized through intra-dimer As···I interactions of 3.607(1) Å. The dimers are further linked by additional As···I contacts of 3.518(1) Å. All these intermolecular As···I contacts are shorter than the sum of the van der Waals radii of arsenic and iodine (3.83 Å) [30]. The shortest contacts between neighboring chains in **1** correspond to the S2···S2 distances of 3.496(2) Å. Compared with the other complexes, compound **2** crystallizes forming a surprisingly large unit cell which consists of 13 crystallographically independent molecules in the asymmetric unit (Table 2 and Figure 4). Moreover, according to the Cambridge Structural Database, there is a relatively small number of structures with such a high number of molecules per asymmetric unit (Z’) with only 30 different structures for Z’ ≥ 13 reported to date [31]. The remaining compounds possess only one molecule in the asymmetric part. Similarly to compound **2**, its chlorido analogue also contains a rare high Z’ structure with 17 molecules in the asymmetric unit [24]. The 13 molecules in compound **2** form 7 crystallographically different dimers through intra-dimer As···Br contacts in the range 3.380(1)–3.586(1) Å. Only one of these units is constructed from two of the same molecules containing As1B atoms. All dimers are held together by additional As···Br distances remaining in the range 3.465(2)–3.656(2) Å. These inter-dimer As···Br contacts are usually slightly longer than the intra-dimer distances. Nevertheless, all aforementioned distances are shorter than the sum of the van der Waals radii (3.68 Å) [30]. Additionally, some As···S contacts (3.567(2)–3.642(2) Å) between molecules of compound **2** are very close to the sum of the respective van der Waals radii (3.65 Å) [30]. The molecular packing in compounds **3** and **4** is essentially the same and consists of dimeric units arranged into a three dimensional net (Figure 5) with similar intra- [3.777(1) (As···I) or 3.698(1) Å (As···Br)] and inter-dimer [3.782(1) (As···I) or 3.714(1) Å (As···Br)] contacts, that remain very close to the sum of the respective van der Waals radii [30]. In addition, the molecules are stabilized by S···C contacts of 3.499(6) and 3.546(2) Å (for compounds **3** and **4**, respectively) between the adjacent chelating rings. The shortest distances between neighboring arsenic atoms in **1** (3.922(1) Å) and **2** (mean 3.945(2) Å; range 3.847(2)–4.004(2)) lay outside the dimers. By contrast, the shortest As···As contacts for compound **3** (4.645(1) Å) and **4** (4.376(1) Å) are placed within the dimeric entities. All these distances fall beyond twice the van der Waals radius of arsenic (3.70 Å) [30].

### 2.3. DFT Modelled Molecular Structures

**Figure 6:**
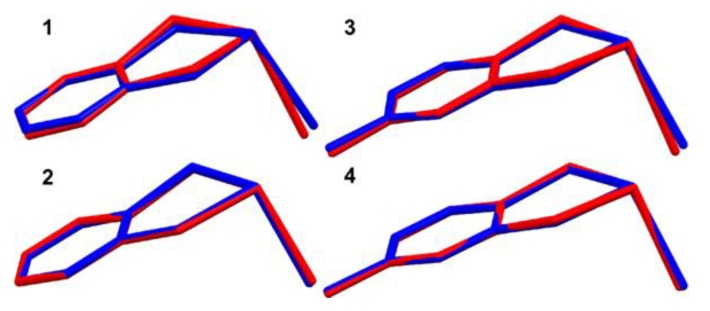
Graphical presentation showing the overlay of the molecular structures derived from DFT calculation (red) and experiment (blue) for AsI(PhS_2_) (**1**) (RMSD = 0.146 Å), AsBr(PhS_2_) (**2**) (RMSD = 0.042 Å), AsI(MePhS_2_) (**3**) (RMSD = 0.111 Å), AsBr(PhS_2_) (**4**) (RMSD = 0.041 Å).

The geometric parameters of the presented arsenic(III) compounds were also optimized by means of various basis sets within B3LYP functional. The smallest mean absolute deviation (MAD) values determined for the chosen bond lengths and angles indicate that satisfactory correlations between the experimental and calculated structures for all compounds are obtained using a LANL2DZ basis set for iodine atom and 6-31+G(d,p) basis set for the remaining elements (see Tables S1–S4). Satisfactory results are also otbained using 6-31G(d,p) and LANL2DZ basis sets for compounds **1** and **3** or 6-31++G(d,p) basis set for compunds **2** and **4**. The most important bond lengths and angles for studied complexes derived from the crystal structure are in a good agreement with calculations presented in Table 1 and Figure 6. In all cases the differences between experimental and modelled bond distances are no longer than 0.05 Å. The largest differences (approximately 0.05 Å) are observed for As–Br bond lengths.

### 2.4. Spectroscopic Characterization of the Arsenic(III) Compounds

The infrared absorption spectra of the studied complexes were compared with the spectra obtained for the free dithiol ligands (Figures S1–S6). The formation of dithiolato arsenic(III) compounds are confirmed through the obtained infrared (IR) spectra by the complete removal of the broad S–H stretching vibrations located at around 2538 cm^−1^ present in the free ligands. Comparing the IR data, compounds **1** with **2**, and **3** with **4** have very similar spectra with only slight shifts between equivalent bands. Almost the entire spectra are dominated by vibrations of the aromatic ring and C–H bonds. The experimentally obtained IR spectra of complexes **1**–**4** were in good agreement with the calculated data (see Figures S2, S3, S5 and S6).

**Figure 7:**
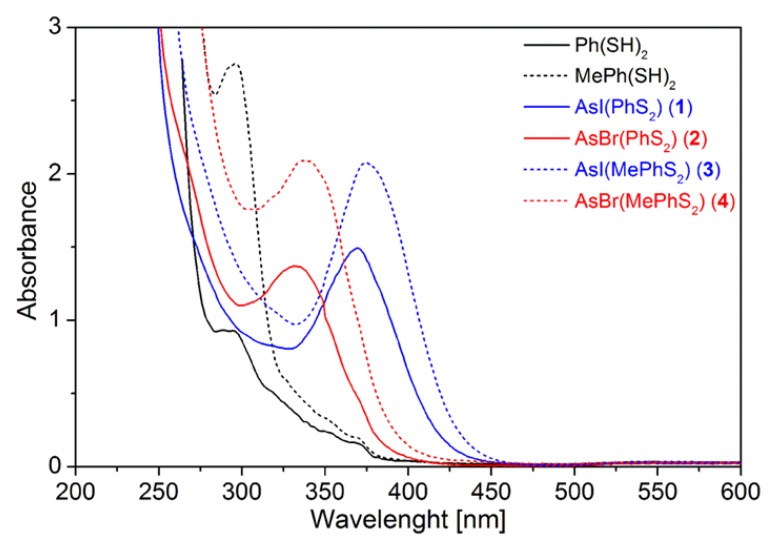
Ultraviolet-visible (UV-Vis) spectra of free dithiol ligands and their arsenic(III) compounds.

**Figure 8:**
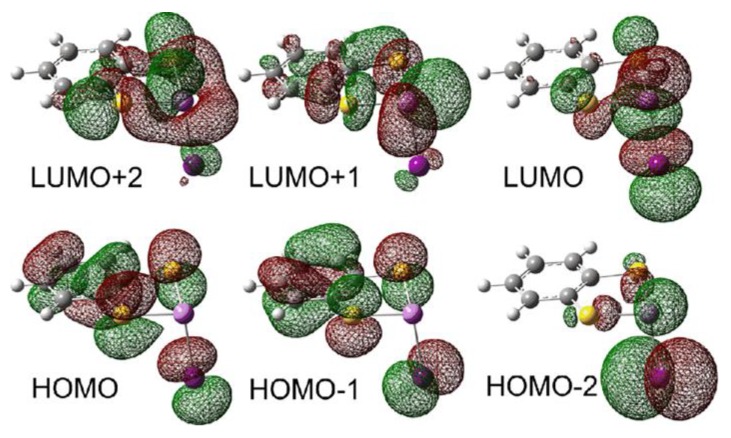
Orbital contours of the lowest energy transitions for **1** as representative of all studied compounds.

In general, UV-Vis spectra of the studied complexes display intense absorptions below 280 nm, accompanied by a lower-intensity band at 370 and 375 nm for iodo or 331 and 336 nm for bromo species (Figure 7). An absorption maximum at 296 nm and a shoulder at about 290 nm are observed for pure toluene-3,4-dithiol and 1,2-benzenedithiol but not after complexation to arsenic(III). Changing the halogen atom from bromine to iodine for the respective dithiolato complex shifts the lower energy band by about 40 nm to higher wavelengths. Furthermore, the addition of a methyl group to 1,2-benzenedithiolato ligand contributes to only a slight shift of the band maximum of the corresponding complex to higher wavelengths. TD-DFT calculated absorption spectra of these compounds in a simulated solvent (dichloromethane) are in good agreement with experimental results (Figure S7). The data presented in Table 3 correspond to the major singlet excitations with the lowest energies. The polarizable continuum model (IEFPCM) was used to model solvation effects during calculations by means of B3LYP method. The calculated transitions are shown in Tables S5–S8. The frontier orbitals of **1**, taken as representative examples for the studied compounds are presented in Figure 8 (compound **1** has very similar MO diagrams to **2**−**4**, see Figure S8). The HOMO and LUMO energy differences are equal to 3.84, 4.33, 3.73 and 4.21 eV for compounds **1**−**4**, respectively. These energy gaps are higher by about 0.5 eV for bromide bearing compounds. In addition, the presence of a methyl group on the bidentate ligand displays a slightly lower energy difference of about 0.1 eV, compared with the analogous complexes bereft of CH_3_ moiety. According to TD-DFT results, shown in Table 3, the lowest energy transitions, corresponding to the major bands above 300 nm, involve mainly the promotion of an electron from HOMO to LUMO (for **1**−**4**) and LUMO+2 (for **2** and **4**). In lesser extent these absorptions are accompanied by HOMO → LUMO+1, HOMO−1 → LUMO and, additionally for **1** and **3**, HOMO−2 → LUMO excitations. As shown in Table 4 the two highest occupied MOs of compounds **1**−**4** are the orbitals of dithiolato and halide ligands with the predominant contribution from the bidentate ligand. The HOMO−2 orbital is located primarily on the halide ion. The lowest occupied MO orbital displays a mixed As/halogen/bidentate ligand character with the highest contribution being from the arsenic atom. In turn, the next two LUMOs (LUMO+1 and LUMO+2) of all compounds are the orbitals of the dithiolato ligand and arsenic. Therefore, the lowest-lying electronic transitions of compounds **1**−**4** have mainly mixed ligand-to-metalloid (bidentate ligand → As) charge transfer and ligand-centered transitions involving dithiolato ligand. In addition, some contributions from ligand-to-ligand (bidentate ligand → monodentate ligand) and halide-to-metalloid (halide → As) charge transfers are also observed.

In the ^13^C NMR spectra of **1** and **2** the signals related to the benzene ring appear as three of six peaks. The higher number of these signals for **3** and **4** is due to the presence of the methyl group on the aromatic ring, which causes all carbons to become magnetically unequivalent. Similarly, the aromatic protons give two (without CH_3_ group) or three signals in the ^1^H NMR spectra. The CH_3_ group is found upfield at 2.4 and 20.8 ppm in the ^1^H and ^13^C NMR spectra, respectively. All registered NMR spectra are shown in Figures S9–S12.

### 2.5. Cytotoxicity

Arsenic trioxide induces apoptosis not only in APL cells [32] but also in many cancer cell lines, such as solid cancer cells like ovarian, esophageal, and prostate carcinomas [33,34,35]. There are also many other As_2_O_3_ sensitive cancer cells, for example small cell lung carcinoma [36], gastric cancer [37], neuroblastoma [38], multiple myeloma [39] and non-APL hematologic malignancies [40]. However, As_2_O_3_ is mainly applied in APL treatment where complete remission is observed in 85−93% patients. The disadvantages in arsenic treatment are its side effects; nevertheless they are usually mild and can be resolved with the appropriate symptomatic treatment or with reduction of As_2_O_3_ dose [32].

In clinical practice the administration of As_2_O_3_ to patients with APL could cause changes associated with an abnormal heart rhythm, which manifests from prolongation of the QT interval on an electrocardiogram diagram [41,42,43]. The study in a mouse model confirmed that arsenic used in concentrations comparable to those applied in humans causes myocardial apoptosis. Analysis of the myocardial function showed that As_2_O_3_ contributes to functional changes in the heart and were accompanied by cardiomyopathy, as determined by histopathological and ultrastructural examination [44].

**Figure 9:**
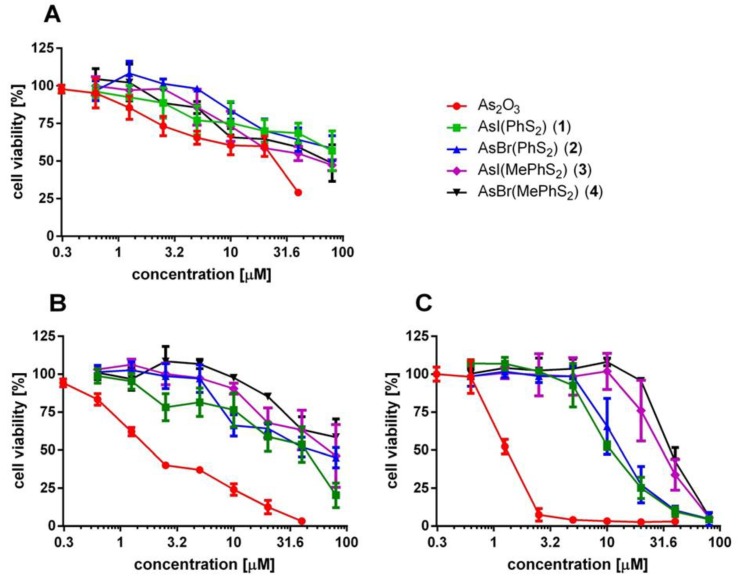
The results of cytotoxicity studies performed on NB4 cells with the use of dithiolato arsenic(III) compounds and As_2_O_3_ after 8 h (**A**), 24 h (B) and 48 h (**C**) of treatment. The data points represent mean ± standard deviation of three independent experiments. Statistical significance was considered when *p* ˂ 0.05 (image B for compound **2**, **3**), *p* < 0.01 (image B for compound **4**).

An important accept of these arsenic complexes should be their comparative mode of action to As_2_O_3_, which would have a smaller influence on myocardial function. In our studies we examined the potency of arsenic(III) compounds **1**–**4**, and arsenic trioxide as an anticancer reference agent, on cell growth of acute promyelocytic leukemia cells, NB4. As shown in Figure 9, all compounds as well as As_2_O_3_ induced cytotoxicity in a time and dose-dependent manner. Our results demonstrate that the viability of the cells treated with compounds **1**–**4** is higher than the viability of cells exposed for As_2_O_3_. Furthermore, based on the cytotoxicity data obtained, after 48 h treatment of the cancer cells, the IC_50_ values were determined. In comparison to the literature, the NB4 cells are relatively sensitive toward As_2_O_3_. Moreover, the estimated IC_50_ value (1.43 µM) is below clinically tolerable doses [36] (Table 5). Our synthesized anticancer agents **1**–**4** exhibited lower cytotoxicity (about 7-fold lower for compounds **1**–**2** and nearly 28-fold lower for compounds **3**–**4**) in comparison to arsenic trioxide. The differences of NB4 cells in sensitivity to synthesized compounds and As_2_O_3_ could be explained by their structural differences. The addition of a methyl group to the benzene ring (**3**–**4** molecules) causes lower reduction in viability of promyelocytic leukemia cells in comparison to compounds **1**–**2**. Moreover, the presence of Br^−^ or I^−^ anions in the structures did not influence the cytotoxicity. However, all newly prepared dithiolato arsenic(III) compounds inhibited cell proliferation, which is important for future investigations of potential applications in radionuclide cancer therapy. Research of these compounds creates new possibilities to connect arsenic with targeting biomolecules and apply similar compounds for nuclear medicine using radioactive arsenic isotopes.

## 3. Materials and Methods

All chemicals and solvents used were purchased from commercial sources. Elemental analyses were performed on an Elementar Vario EL III analyzer (Hanau, Germany). UV-Vis spectra in CH_2_Cl_2_ solutions were measured on a Thermo Scientific Evolution 600 spectrometer (Madison, WI, USA). Infrared absorption spectra in the range 4000–370 cm^−1^ at a resolution of 2 cm^−1^ were registered with a Thermo Scientific Nicolet iS10 FT-IR spectrometer (Madison, WI, USA) using KBr pellets. ^1^H and ^13^C NMR spectra using CDCl_3_ as a solvent were recorded with a Varian Unity Plus 500 MHz spectrometer (Palo Alto, CA, USA). ESI-MS spectra were recorded with an Agilent Technologies 6530 Accurate-Mass Q-TOF LC/MS device (Santa Clara, CA, USA) equipped with a Zorbax Extend-C18 column (2.1 × 50 mm, 1.8 μm) working with a gradient of 5–100% acetonitrile within 3.5 min and a flow rate of 0.6 mL/min. High-performance liquid chromatography (HPLC) was performed using ELITE LaChrom (Hitachi, Tokyo, Japan) system with an L-2310 pump coupled to L-2455 diode array detector and L-2350 column oven. An Aeris Peptide column (3.6u XB-C18 150 × 4.6 mm) eluting at a flow rate of 1 mL/min was used. The gradient elution system contained 0.1% trifluoroacetic acid in deionized water (eluent A) and acetonitrile (eluent B). The gradient started with 95% A/5% B for 5 min; this was increased to 100% B over the next 15 min and held at 100% B for an additional 10 min. Then, the gradient parameters were returned to the initial conditions over the 5 min. HPLC grade solvents were degassed by ultrasonification for about 20 min just before use in chromatography measurements.

### 3.1. Preparation of the Complexes

All complexes were prepared in a similar way by the methods described in [25]. To 1 mmol of ligand (1,2-benzenedithiol or toluene-3,4-dithiol) dissolved in CHCl_3_ saturated with nitrogen, an equimolar amount of arsenic(III) compound (AsBr_3_ or AsI_3_) was added. The reaction mixture was heated and stirred under reflux for 5 h. Next, the solution was concentrated under reduced pressure and placed in a fridge. The crystals of each compound were isolated from the reaction mixture after a week.

#### 3.1.1. Iodo-(benzene-1,2-dithiolato-S,S’)-arsenic(III)—AsI(PhS_2_) (**1**)

Yield: 0.163 g (47.7%). Anal. calc. for C_6_H_4_S_2_AsI: C, 21.07; H, 1.18; S, 18.75; found C, 21.08; H, 1.18; S 18.8 %. UV-Vis (CH_2_Cl_2_) *λ*_max_/nm (ε/M^−1^·cm^−1^): 370 (6000). IR (KBr) ν_max_/cm^−1^: 1443w, 1421m, 1248w, 1135w, 933w, 846w, 741vs, 660w, 465w, 429w, 407w, 390s. ^1^H NMR (500.20 MHz, CDCl_3_) *δ*/ppm = 7.59 (dd, *J* = 6.0, 3.0 Hz, 2H, Ar–H), 7.24 (dd, *J* = 6.0, 3.5 Hz, 2H, Ar–H). ^13^C NMR (125.79 MHz, CDCl_3_) *δ*/ppm = 140.92 (2C, Ar), 127.18 (2C, Ar), 126.23 (2C, Ar). ESI-MS *m/z* = 214.8966 [M − I + H]^+^ (calc. 214.8965). HPLC R_t_ = 9.7 min.

#### 3.1.2. Bromo-(benzene-1,2-dithiolato-S,S’)-arsenic(III)—AsBr(PhS_2_) (**2**)

Yield: 0.122 g (41.3%). Anal. calc. for C_6_H_4_S_2_AsBr: C, 24.42; H, 1.37; S, 21.74; found C, 24.47; H, 1.35; S, 21.75%. UV-Vis (CH_2_Cl_2_) *λ*_max_/nm (ε/M^−1^·cm^−1^): 331 (4150). IR (KBr) ν_max_/cm^−1^: 1445m, 1422m, 1250w, 1135w, 1106w, 934w, 742vs, 661w, 467w, 429w, 409w, 396m. ^1^H NMR (500.20 MHz, CDCl_3_) *δ*/ppm = 7.62 (dd, *J* = 5.5, 3.3 Hz, 2H, Ar–H), 7.24 (dd, *J* = 6.0, 3.5 Hz, 2H, Ar–H). ^13^C NMR (125.79 MHz, CDCl_3_) *δ*/ppm = 139.92 (2C, Ar), 127.01 (2C, Ar), 126.20 (2C, Ar). ESI-MS *m/z* = 214.8966 [M − Br + H]^+^ (calc. 214.8965). HPLC R_t_ = 9.7 min.

#### 3.1.3. Iodo-(toluene-3,4-dithiolato-S,S’)-arsenic(III)—AsI(MePhS_2_) (**3**)

Yield: 0.207 g (56.7%). Anal. calc. for C_7_H_6_S_2_AsI: C, 23.61; H, 1.70; S, 18.01; found C, 23.76; H, 1.74; S 18.00%. UV-Vis (CH_2_Cl_2_) *λ*_max_/nm (ε/M^−1^·cm^−1^): 375 (6300). IR (KBr) ν_max_/cm^−1^: 1459m, 1380w, 1253w, 855w, 805vs, 693w, 684w, 637w, 536m, 486w, 448m, 427w, 400m, 398m. ^1^H NMR (500.20 MHz, CDCl_3_) *δ*/ppm = 7.47 (d, *J* = 8.0 Hz, 1H, Ar–H), 7.40 (s, 1H, Ar–H), 7.05 (d, *J* = 8.5 Hz, 1H, Ar–H), 2.37 (s, 3H, CH_3_). ^13^C NMR (125.79 MHz, CDCl_3_) *δ*/ppm = 141.10 (1C, Ar), 137.63 (1C, Ar), 136.59 (1C, Ar), 127.50 (1C, Ar), 127.40 (1C, Ar), 126.76 (1C, Ar), 20.80 (1C, CH_3_). ESI-MS *m/z* = 228.9121 [M − I + H]^+^ (calc. 228.9121). HPLC R_t_ = 10.3 min.

#### 3.1.4. Bromo-(toluene-3,4-dithiolato-S,S’)-arsenic(III)—AsBr(MePhS_2_) (**4**)

Yield: 0.124 g (40.1%). Anal. calc. for C_7_H_6_S_2_AsBr: C, 27.20; H, 1.96; S, 20.75; found C, 27.20; H, 1.97; S 20.80%. UV-Vis (CH_2_Cl_2_) *λ*_max_/nm (ε/M^−1^·cm^−1^): 336 (4200). IR (KBr) ν_max_/cm^−1^: 2912w br, 1460m, 1381w, 1254w, 856w, 806vs, 536w, 486w, 449m, 428w, 403m, 398w. ^1^H NMR (500.20 MHz, CDCl_3_) *δ*/ppm = 7.48 (d, *J* = 8.0 Hz, 1H, Ar–H), 7.42 (s, 1H, Ar–H), 7.05 (d, *J* = 8.0 Hz, 1H, Ar–H), 2.37 (s, 3H, CH_3_). ^13^C NMR (125.79 MHz, CDCl_3_) *δ*/ppm = 140.07 (1C, Ar), 136.60 (1C, Ar), 136.57 (1C, Ar), 127.38 (1C, Ar), 127.32 (1C, Ar), 126.60 (1C, Ar), 20.77 (1C, CH_3_). ESI-MS *m/z* = 228.9121 [M − Br + H]^+^ (calc. 228.9121). HPLC R_t_ = 10.4 min.

### 3.2. X-ray Crystallography

Diffraction data of suitable single crystals of studied arsenic(III) compounds were collected at 100 K on a Rigaku SuperNova (dual source) four circle diffractometer equipped with an Eos CCD detector (Oxford, UK). The measurements were performed using a mirror-monochromated Mo or Cu Kα radiation (λ = 0.71073 or 1.54184 Å) from a microfocus Mova or Nova X-ray source, respectively. All required procedures including data collection, data reduction and multi-scan absorption corrections were performed using CrysAlis PRO software (version 1.171.38.41, Oxford, UK). The structures were solved by direct methods and refined by the full matrix least-squares technique on F^2^ data. All non-hydrogen atoms were refined anisotropically while the hydrogen atoms were placed in calculated positions and refined isotropically using standard parameters. All calculations were performed using SHELX programs [45] integrated with the OLEX2 crystallographic software [46]. The MERCURY program [47] was applied for graphical representation of the crystal structures. Selected crystallographic parameters and refinement details are presented in Table 2.

### 3.3. Computational Details

The structures obtained for arsenic(III) compounds from the X-ray measurements were used as the initial geometries in the optimization of their molecular structures. The ground-state geometries of these complexes were modeled using the DFT method with B3LYP hybrid functional [48,49]. The results obtained for a few different double- and triple-ζ valence basis sets were collected and compared (Tables S1–S4). The LANL2DZ basis set [50] was applied for iodine atom when using double-ζ valence basis sets. Vibrational frequency calculations were performed to verify the minimum energy states. The calculated harmonic frequencies were scaled by 0.97. Based on optimized structures, time-dependent DFT [51] calculations with the B3LYP functional and 6-31+G(d,p) basis set including the LANL2DZ basis set for an iodine atom were carried out to obtain absorption spectra. The solvent effect of dichloromethane was modeled using the polarizable continuum model (IEFPCM) [52]. All calculations were carried out with the Gaussian 09 program package [53]. The GaussView 5.0 [53] and GaussSum 3.0 [54] programs were employed for visualization of the molecular orbitals and determination of the percent contributions of atoms and atom groups to each of the MOs.

### 3.4. Cytotoxicity Studies

Human acute promyelocytic leukemia cells, NB4 (purchased from DSMZ company, Braunschweig, Germany) were cultured in RPMI 1640 Medium (Biological Industries, Beit-Haemek, Israel), supplemented with 10% of FBS (Biological Industries, Israel) and 1% of antibiotics (Biological Industries, Israel) at 37 °C in a humidified atmosphere of 5% CO_2_ in the air. The effects of new potential cytotoxic drugs on cell viability were determined by MTS assay as previously described [55]. Briefly, the cells were seeded in 96-well plates (3000 cells/well) and incubated over-night at 37 °C in 5% CO_2_. After 24 h, the cells were treated with compounds **1**−**4** at different concentrations varied from 0.625 to 80 µM. As_2_O_3_ was used as a reference compound. After 8 h, 24 h and 48 h of incubation, 20 μL aliquots of MTS solution (CellTiter 96^®^ AQueous reagent, Promega, Mannheim, Germany) were added to each well and re-incubated for 2 h at 37 °C. Then, the absorbance was measured at 492 nm, on microplate reader (Apollo-1, LB913, Berthold, Bad Wildbad, Germany). The percentage of cell viability was calculated from the control group. All treatments were conducted in sixplicate and the experiments were repeated thrice.

### 3.5. Statistical Analysis

The cytotoxicity experiments (MTS assay) were performed at least in triplicate. To assess the differences between compounds **1**−**4** and control (As_2_O_3_) one-way analysis of variance (ANOVA), followed by Dunnett’s multiple comparisons test was applied. Statistical analysis was performed using GraphPad Prism 8.0 software (GraphPad Software Inc., San Diego, CA, USA). Data were presented as mean ± standard deviation (SD) and the results were considered statistically significant when *p*-value was <0.05.

## 4. Conclusions and Summary

In this work four new arsenic(III) complexes (**1**–**4**) with heavy halide ions (bromide and iodide) and two dithiolato ligands derived from 1,2-benzenedithiol and toluene-3,4-dithiol were synthesized and characterized using conventional physico-chemical methods. Among the compounds obtained, the AsBr(PhS_2_) complex (**2**) has a structure with a rare 13 molecules in the asymmetric unit. The complexes **1** and **2** possessing the 1,2-benzenedithiolato ligand exhibited, after 48 h of treatment, 4-fold higher in vitro cytotoxicity (IC_50_ ≈ 10 µM) toward human acute promyelocytic leukemia cancer cells (NB4) than compounds **3** and **4** with toluene-3,4-dithiolato ligand (IC_50_ ≈ 40 µM). The replacement of an iodide anion with a bromide anion in the studied complexes does not affect the change in their toxicity. Although their toxicity is lower compared to As_2_O_3_ (IC_50_ = 1.4 µM), a popular chemotherapeutic agent, it still seems to be suitable for cancer treatment. The studies presented show that prepared compounds are potentially promising anticancer agents worthy of further investigation. The huge advantage of the studied compounds is also the possibility of exchanging stable arsenic for radioisotope ^77^As, which could significantly increase the therapeutic effect, especially since there is a strong synergistic effect between chemo and radiotherapy [56].

## Figures and Tables

**Table 1:** Selected bond lengths (Å) and angles (°) of complexes **1**−**4** obtained from experiment and DFT calculations (B3LYP/6-31+G(d,p);LANL2DZ) (X = I1 (**1** and **3**) and Br1 (**2** and **4**)). * In the case of **2** the experimental data are given as mean values from 13 molecules.

Compound	1		2		3		4	
	Exp.	Calc.	Exp.*	Calc.	Exp.	Calc.	Exp.	Calc.
*Bond lengths*								
As1-S1	2.218(1)	2.252	2.212(3)	2.243	2.216(1)	2.250	2.208(1)	2.245
As1-S2	2.227(1)	2.252	2.210(3)	2.246	2.212(1)	2.252	2.205(1)	2.242
As1-X	2.730(1)	2.700	2.467(1)	2.421	2.699(1)	2.704	2.469(1)	2.423
S1-C1	1.764(5)	1.779	1.759(9)	1.778	1.752(4)	1.779	1.757(2)	1.779
S2-C2	1.763(4)	1.779	1.759(9)	1.779	1.762(5)	1.779	1.761(2)	1.779
*Angles*								
X-As1-S1	98.69(4)	103.27	100.81(7)	101.91	103.32(4)	103.40	102.96(1)	101.66
X-As1-S2	106.10(4)	103.29	100.83(7)	101.57	101.31(4)	103.22	100.86(1)	101.76
S1-As1-S2	92.97(5)	92.09	93.20(9)	93.10	93.29(5)	92.15	93.38(2)	93.22

**Table 2:** Crystallographic data and structure refinement parameters of the compounds **1**−**4**.

Compound	1	2	3	4
Chemical formula	C_6_H_4_AsIS_2_	C_6_H_4_AsBrS_2_	C_7_H_6_AsIS_2_	C_7_H_6_AsBrS_2_
Formula weight	342.03	295.04	356.06	309.07
Temperature (K)	100(2)	100(2)	100(2)	100(2)
*λ* [Cu or Mo K*α*] (Å)	0.71073	1.54184	1.54184	0.71073
Crystal system	triclinic	triclinic	monoclinic	monoclinic
Space group	*P* $\bar{1}$	*P* $\bar{1}$	*P* 2_1_/*c*	*P* 2_1_/*c*
*a* (Å)	4.4828(4)	16.1708(7)	10.0491(3)	9.77969(10)
*b* (Å)	9.3077(9)	16.9940(6)	12.1431(4)	12.05914(14)
*c* (Å)	11.4679(11)	21.9286(9)	8.1564(3)	8.09708(9)
*α* (°)	68.272(9)	111.907(4)	90.00	90.00
*β* (°)	87.667(7)	92.321(4)	104.407(3)	105.2034(11)
*γ* (°)	81.062(8)	100.773(4)	90.00	90.00
Volume (Å)	439.03(7)	5452.1(4)	964.00(5)	921.504(17)
*Z*	2	26	4	4
*D*_calc_ (g·cm^−3^)	2.587	2.336	2.453	2.228
µ (mm^−1^)	7.783	15.056	33.429	8.407
*F* (000)	316	3640	664	592
Crystal size (mm)	0.10 × 0.06 × 0.03	0.20 × 0.12 × 0.08	0.20 × 0.18 × 0.06	0.18 × 0.16 × 0.08
Reflections collected	5438	46,507	9744	49,374
Independent reflections	2112	18,509	1780	2445
*R*_int_	0.0688	0.0492	0.0618	0.0365
Data/restraints/parameters	2112/0/91	18,509/0/1141	1780/0/101	2445/0/101
GOF(*F*^2^)	1.077	1.071	1.077	1.090
Final *R* indices [*I* > 2σ(*I*)]	*R*_1_ = 0.0389 *wR*_2_ = 0.0871	*R*_1_ = 0.0669 *wR*_2_ = 0.1998	*R*_1_ = 0.0386 *wR*_2_ = 0.1066	*R*_1_ = 0.0172 *wR*_2_ = 0.0374
R indices (all data)	*R*_1_ = 0.0455 *wR*_2_ = 0.0913	*R*_1_ = 0.0974 *wR*_2_ = 0.2440	*R*_1_ = 0.0387 *wR*_2_ = 0.1068	*R*_1_ = 0.0184 *wR*_2_ = 0.0378
Largest difference in peak/hole (e·Å^−3^)	1.548/−1.776	1.636/−3.382	2.570/−1.781	1.365/−0.577

**Table 3:** The highest wavelength absorption data from experiments and calculations (TD-DFT) for the studied arsenic(III) compounds.

Compound	Calculated			Experimental
	λ (nm)	Oscillator Strength	Main Components	λ (nm)
**1**	381.49	0.1485	H→L (94.2%)	370
	355.05	0.0055	H − 1→L (94.2%)	
	342.02	0.0086	H − 2→L (98.7%)	
	332.22	0.0031	H→L + 1 (95.6%)	
**2**	345.16	0.0686	H→L (88.4%)	331
			H→L + 2 (9.6%)	
	310.46	0.0060	H − 1→L (85.8%)	
			H→L + 1 (8.7%)	
	301.80	0.0640	H→L + 2 (84.6%)	
			H→L (8.3%)	
**3**	392.75	0.1447	H→L (94.9%)	375
	358.86	0.0082	H − 1→L (93.2%)	
	342.56	0.0079	H − 2→L (98.6%)	
	338.15	0.0041	H→L + 1 (95.6%)	
**4**	354.59	0.0676	H→L (89.2%)	336
			H→L + 2 (9.0%)	
	314.95	0.0052	H − 1→L (87.0%)	
			H→L + 1 (5.2%)	
	308.52	0.0700	H→L + 2 (84.7%)	
			H→L (7.8%)	

**Table 4:** DFT (B3LYP/IEFPCM) calculated compositions of selected highest occupied and lowest unoccupied molecular orbitals of studied compounds in dichloromethane, expressed in terms of composing fragments (X – I (for **1** and **3**) or Br (**2** and **4**); L_B_—bidentate ligand: PhS_2_ (**1** and **3**) or MePhS_2_ (**2** and **4**)).

Compound	MO	As	X	L_B_
**1**	LUMO + 1	64	2	34
	LUMO	47	30	23
	HOMO	3	16	81
	HOMO − 1	0	18	82
	HOMO − 2	10	84	6
**2**	LUMO + 2	48	3	49
	LUMO + 1	69	1	30
	LUMO	46	27	27
	HOMO	3	11	86
	HOMO − 1	0	5	95
**3**	LUMO + 1	64	2	34
	LUMO	48	29	23
	HOMO	2	15	83
	HOMO − 1	0	15	85
	HOMO − 2	10	84	6
**4**	LUMO + 2	48	3	49
	LUMO + 1	69	2	29
	LUMO	47	27	26
	HOMO	3	9	88
	HOMO − 1	0	5	95

**Table 5:** Concentrations of different anti-cancer agents that inhibit 50% of NB4 cell growth, after 48 h of treatment.

Compound	IC_50_ [µM]
As_2_O_3_	1.43 ± 0.07
**1**	10.05 ± 0.31
**2**	10.28 ± 0.17
**3**	39.71 ± 0.15
**4**	39.87 ± 0.08

## Supplementary Materials

CCDC 1948452-1948455 contain the supplementary crystallographic data for this paper. These data can be obtained free of charge from the CCDC via http://www.ccdc.cam.ac.uk/data_request/cif.

The other Supplementary Materials are available online at https://www.mdpi.com/1420-3049/24/21/3865/s1: Tables S1–S4: Comparison of the experimentally obtained and calculated (DFT/B3LY P/6-31+G(d,p);LANL2DZ for I) bond lengths and angles for compounds **1**–**4** (MAD means the mean absolute deviation), Figure S1: IR spectrum of 1,2-benzenedithiol (Ph(SH)_2_), Figure S2. IR spectrum of AsI(PhS_2_) (**1**), Figure S3: IR spectrum of AsBr(PhS_2_) (**2**), Figure S4: IR spectrum of toluene-3,4-dithiol (MePh(SH)_2_), Figure S5: IR spectrum of AsI(MePhS_2_) (**3**), Figure S6: IR spectrum of AsBr(MePhS_2_) (**4**), Figure S7: Experimental (black line) and TD-DFT simulated (red vertical lines) UV-Vis absorption spectra of the studied complexes in CH_2_Cl_2_ solution, Figure S8: Unoccupied and occupied orbital contours relative to the lower energy transitions for compounds **1**–**4**, Tables S5–S8: Calculated transitions for compounds **1**–**4**, Figure S9: (a) ^1^H NMR and (b) ^13^C NMR spectra of AsI(PhS_2_) (**1**), Figure S10: (a) ^1^H NMR and (b) ^13^C NMR spectra of AsBr(PhS_2_) (**2**), Figure S11: (a) ^1^H NMR and (b) ^13^C NMR spectra of AsI(MePhS_2_) (**3**), Figure S12: (a) ^1^H NMR and (b) ^13^C NMR spectra of AsBr(MePhS_2_) (**4**).
