# Supplementary material for: 1,2-Benzenedithiol and Toluene-3,4-dithiol Arsenic(III) Complexes—Synthesis, Structure, Spectroscopic Characterization and Toxicological Studies
Source: Molecules. 2019 Oct 26;24(21):3865. doi: 10.3390/molecules24213865 (PMC6864545; doi:10.3390/molecules24213865)
Supplement: Supplementary file 1 [file molecules-24-03865-s001.pdf]

1,2-Benzenedithiol and toluene-3,4-dithiol arsenic(III) complexes - synthesis, structure, spectroscopic characterization and toxicological studies

Monika Lyczko, Krzysztof Lyczko, Agnieszka Majkowska-Pilip, Aleksander Bilewicz

Institute of Nuclear Chemistry and Technology, Dorodna 16, 03-195 Warsaw, Poland

Supplementary Materials

Legends

Tables S1-S4. Comparison of the experimentally obtained and calculated (DFT/B3LYP/6-31+G(d,p);LANL2DZ for I) bond lengths and angles for compounds **1-4** (MAD means the Mean Absolute Deviation).
Figure S1. IR spectrum of 1,2-benzenedithiol ($Ph(SH)_2$).
Figure S2. IR spectrum of $AsI(PhS_2)$ (**1**).
Figure S3. IR spectrum of $AsBr(PhS_2)$ (**2**).
Figure S4. IR spectrum of toluene-3,4-dithiol ($MePh(SH)_2$).
Figure S5. IR spectrum of $AsI(MePhS_2)$ (**3**).
Figure S6. IR spectrum of $AsBr(MePhS_2)$ (**4**).
Figure S7. Experimental (black line) and TD-DFT simulated (red vertical lines) UV-vis absorption spectra of the studied complexes in CH_2Cl_2 solution.
Figure S8. Unoccupied and occupied orbital contours relative to the lower energy transitions for compounds **1-4**.

Tables S5-S8. Calculated transitions for compounds **1-4**.
Figure S9 a) ^1H NMR and **b)** ^{13}C NMR spectra of $AsI(PhS_2)$ (**1**).
Figure S10 a) ^1H NMR and **b)** ^{13}C NMR spectra of $AsBr(PhS_2)$ (**2**).
Figure S11 a) ^1H NMR and **b)** ^{13}C NMR spectra of $AsI(MePhS_2)$ (**3**).
Figure S12 a) ^1H NMR and **b)** ^{13}C NMR spectra of $AsBr(MePhS_2)$ (**4**).

Table S1. Comparison of the experimentally obtained and calculated (DFT/B3LYP/6-31+G(d,p);LANL2DZ for I) bond lengths and angles for compound **1** (MAD means the Mean Absolute Deviation).

**AsI(PhS₂) (1)**		**B3LYP 6-31G(d,p) LANL2DZ**		**B3LYP 6-31+G(d,p) LANL2DZ**		**B3LYP 6-31++G(d,p) LANL2DZ**		**B3LYP 6-311++G(d,p) LANL2DZ**		**B3LYP def2-TZVP ecp**	
	**exp**	**calc**	**∣exp-calc∣**	**calc**	**∣exp-calc∣**	**calc**	**∣exp-calc∣**	**calc**	**∣exp-calc∣**	**calc**	**∣exp-calc∣**
As-I1	2.730	2.713	0.017	2.700	0.030	2.783	0.053	2.753	0.023	2.660	0.070
As-S1	2.218	2.251	0.033	2.252	0.034	2.353	0.135	2.334	0.116	2.245	0.027
As-S2	2.227	2.250	0.023	2.252	0.025	2.352	0.125	2.334	0.107	2.245	0.018
S1-C1	1.764	1.780	0.016	1.779	0.015	1.842	0.078	1.842	0.078	1.766	0.002
S2-C2	1.763	1.780	0.017	1.779	0.016	1.842	0.079	1.842	0.079	1.766	0.003
**MAD**			**0.021**		**0.024**		**0.094**		**0.081**		**0.024**
I1-As-S1	98.69	103.32	4.63	103.27	4.58	103.85	5.16	104.52	5.83	103.59	4.90
I1-As-S2	106.10	103.34	2.76	103.29	2.81	103.87	2.23	104.56	1.54	103.61	2.49
S1-As-S2	92.97	92.16	0.81	92.09	0.88	91.54	1.43	92.02	0.95	91.71	1.26
**MAD**			**2.73**		**2.76**		**2.94**		**2.77**		**2.88**

Table S2. Comparison of the experimentally obtained and calculated (DFT/B3LYP/6-31+G(d,p)) bond lengths and angles for compound **2** (MAD means the Mean Absolute Deviation). * Experimental data are given as mean values taken from 13 molecules of **2**.

**AsBr(PhS₂) (2)**		**B3LYP 6-31G(d,p)**		**B3LYP 6-31+G(d,p)**		**B3LYP 6-31++G(d,p)**		**B3LYP 6-311++G(d,p)**		**B3LYP def2-TZVP ecp**	
	**exp***	**calc**	**|exp-calc|**	**calc**	**|exp-calc|**	**calc**	**|exp-calc|**	**calc**	**|exp-calc|**	**calc**	**|exp-calc|**
As-Br1	2.467	2.395	0.072	2.421	0.046	2.422	0.045	2.442	0.025	2.433	0.034
As-S1	2.212	2.251	0.039	2.243	0.031	2.245	0.033	2.260	0.048	2.243	0.031
As-S2	2.210	2.251	0.041	2.246	0.036	2.245	0.035	2.260	0.050	2.243	0.033
S1-C1	1.759	1.784	0.025	1.778	0.019	1.778	0.019	1.780	0.021	1.767	0.008
S2-C2	1.759	1.784	0.025	1.779	0.020	1.778	0.019	1.780	0.021	1.767	0.008
**MAD**			**0.040**		**0.030**		**0.030**		**0.033**		**0.023**
Br1-As-S1	100.81	101.16	0.35	101.91	1.10	101.81	1.00	102.98	2.17	102.89	2.08
Br1-As-S2	100.83	101.17	0.34	101.57	0.74	101.79	0.96	102.98	2.15	102.89	2.06
S1-As-S2	93.20	92.49	0.71	93.1	0.10	93.24	0.04	91.85	1.35	91.85	1.35
**MAD**			**0.47**		**0.65**		**0.67**		**1.89**		**1.83**

Table S3. Comparison of the experimentally obtained and calculated (DFT/B3LYP/6-31+G(d,p);LANL2DZ for I) bond lengths and angles for compound **3** (MAD means the Mean Absolute Deviation).

**AsI(MePhS$_2$) (3)**		**B3LYP 6-31G(d,p) LANL2DZ**		**B3LYP 6-31+G(d,p) LANL2DZ**		**B3LYP 6-31++G(d,p) LANL2DZ**		**B3LYP 6-311++G(d,p) LANL2DZ**		**B3LYP def2-TZVP ecp**	
	**exp**	**calc**	**ǀexp-calcǀ**	**calc**	**ǀexp-calcǀ**	**calc**	**ǀexp-calcǀ**	**calc**	**ǀexp-calcǀ**	**calc**	**ǀexp-calcǀ**
As-I1	2.699	2.716	0.017	2.704	0.005	2.787	0.088	2.757	0.058	2.663	0.036
As-S1	2.216	2.249	0.033	2.25	0.034	2.351	0.135	2.333	0.117	2.243	0.027
As-S2	2.212	2.250	0.038	2.252	0.040	2.352	0.140	2.334	0.122	2.245	0.033
S1-C1	1.752	1.779	0.027	1.779	0.027	1.841	0.089	1.841	0.089	1.766	0.014
S2-C2	1.762	1.780	0.018	1.779	0.017	1.842	0.080	1.842	0.080	1.766	0.004
**MAD**			**0.027**		**0.025**		**0.106**		**0.093**		**0.023**
I1-As-S1	103.32	103.42	0.10	103.40	0.08	103.97	0.65	104.63	1.31	103.74	0.42
I1-As-S2	101.31	103.29	1.98	103.22	1.91	103.82	2.51	104.49	3.18	103.53	2.22
S1-As-S2	93.29	92.20	1.09	92.15	1.14	91.58	1.71	92.08	1.21	91.75	1.54
**MAD**			**1.06**		**1.04**		**1.62**		**1.90**		**1.39**

Table S4. Comparison of the experimentally obtained and calculated (DFT/B3LYP/6-31+G(d,p)) bond lengths and angles for compound **4** (MAD means the Mean Absolute Deviation).

**AsBr(MePhS2) (4)**		**B3LYP 6-31G(d,p)**		**B3LYP 6-31+G(d,p)**		**B3LYP 6-31++G(d,p)**		**B3LYP 6-311++G(d,p)**		**B3LYP def2-TZVP ecp**	
	**exp**	**calc**	**|exp-calc|**	**calc**	**|exp-calc|**	**calc**	**|exp-calc|**	**calc**	**|exp-calc|**	**calc**	**|exp-calc|**
As-Br1	2.469	2.397	0.072	2.423	0.046	2.424	0.045	2.445	0.024	2.435	0.034
As-S1	2.208	2.250	0.042	2.245	0.037	2.244	0.036	2.259	0.051	2.242	0.034
As-S2	2.205	2.251	0.046	2.242	0.037	2.244	0.039	2.260	0.055	2.244	0.039
S1-C1	1.757	1.784	0.027	1.779	0.022	1.778	0.021	1.780	0.023	1.767	0.010
S2-C2	1.761	1.784	0.023	1.779	0.018	1.779	0.018	1.780	0.019	1.767	0.006
**MAD**			**0.042**		**0.032**		**0.032**		**0.034**		**0.025**
Br1-As-S1	102.96	101.24	1.72	101.66	1.30	101.85	1.11	103.12	0.16	103.04	0.08
Br1-As-S2	100.86	101.12	0.26	101.76	0.90	101.79	0.93	102.94	2.08	102.84	1.98
S1-As-S2	93.38	92.53	0.85	93.22	0.16	93.32	0.06	91.88	1.50	91.88	1.50
**MAD**			**0.94**		**0.79**		**0.70**		**1.25**		**1.19**

Figure S1.

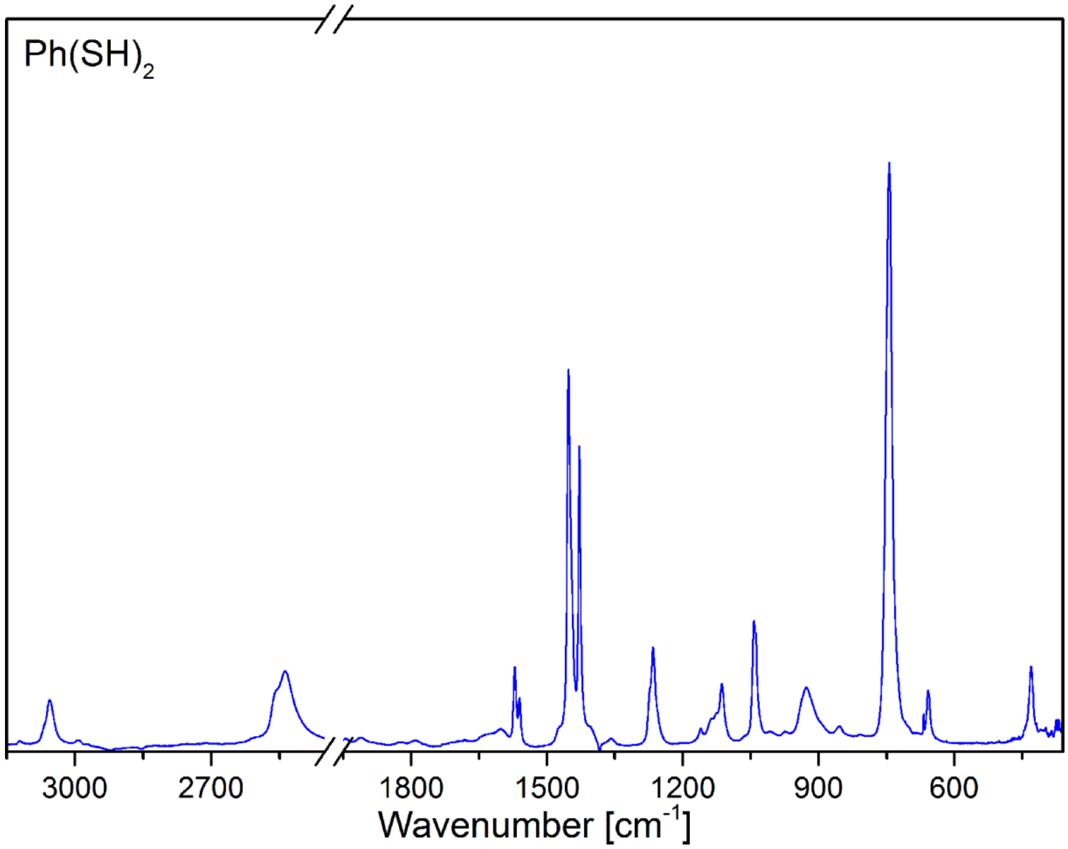

Figure S2.

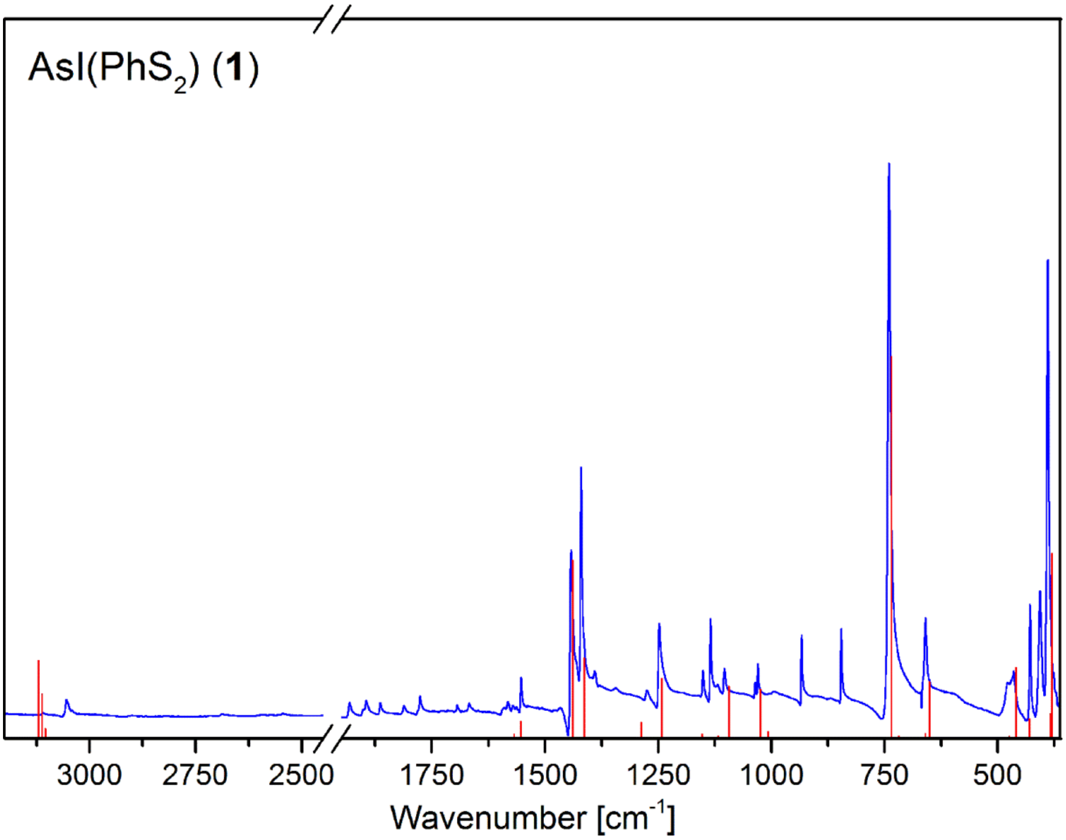

AsI(PhS$_2$) (**1**)
3000
2750
2500
1750
1500
1250
1000
750
500
Wavenumber [cm^{-1}]

Figure S3.

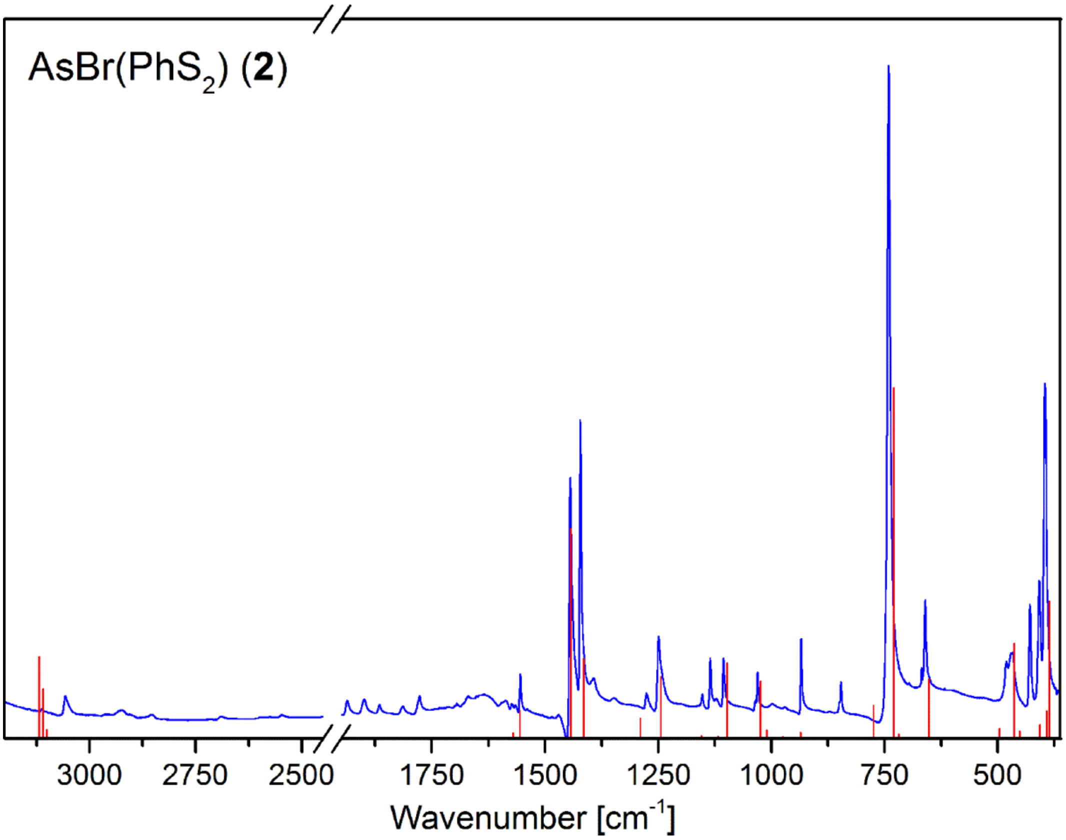

AsBr(PhS$_2$) (**2**)
3000
2750
2500
1750
1500
1250
1000
750
500
Wavenumber [cm^{-1}]

Figure S4.

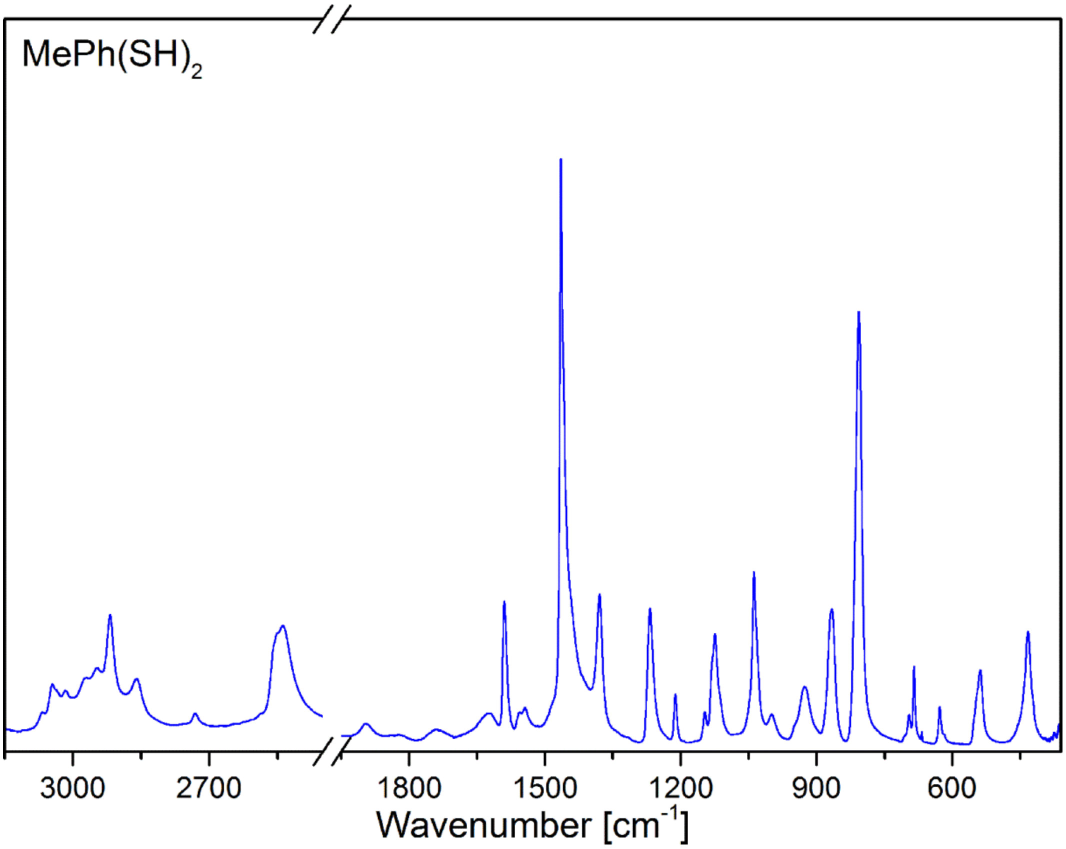

Figure S5.

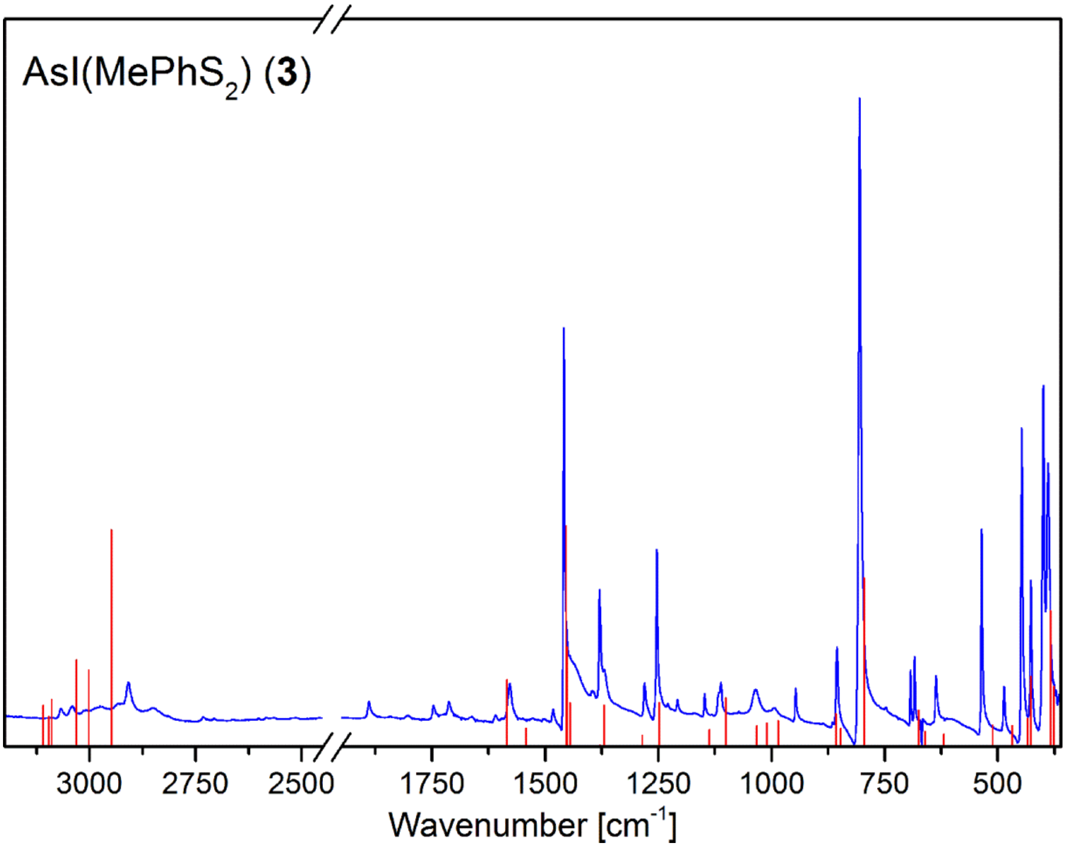

AsI(MePhS$_2$) (**3**)
3000
2750
2500
1750
1500
1250
1000
750
500
Wavenumber [cm^{-1}]

Figure S6.

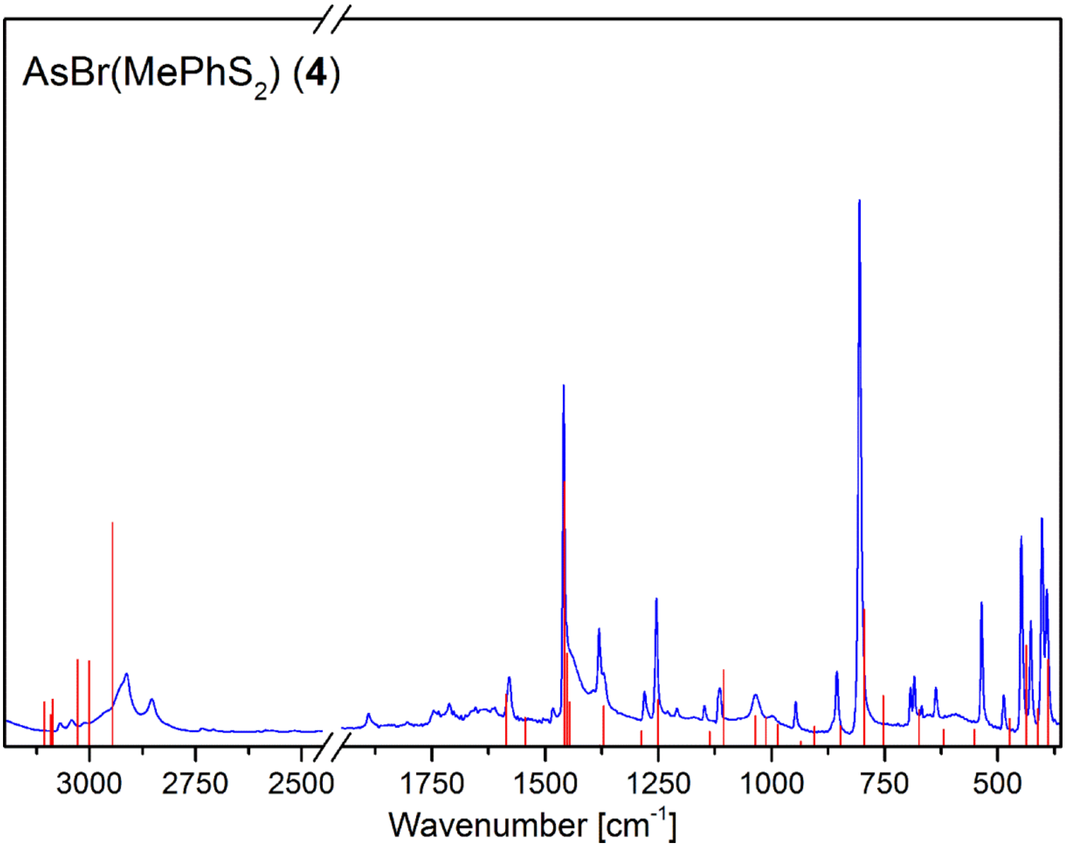

AsBr(MePhS$_2$) (**4**)
3000
2750
2500
1750
1500
1250
1000
750
500
Wavenumber [cm^{-1}]

Figure S7.

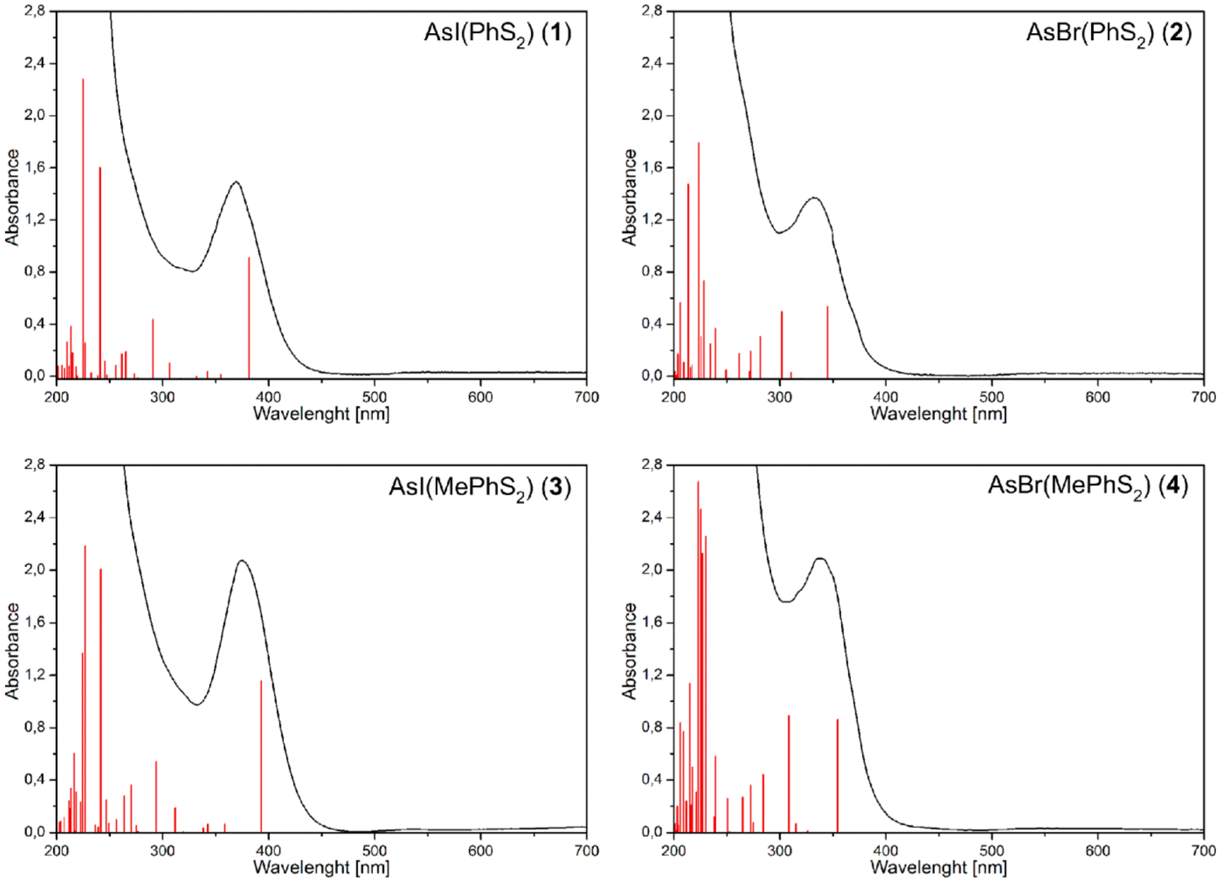
AsI(PhS$_2$) (**1**)
AsBr(PhS$_2$) (**2**)
AsI(MePhS$_2$) (**3**)
AsBr(MePhS$_2$) (**4**)
Absorbance
Wavelenght [nm]
2,8
2,4
2,0
1,6
1,2
0,8
0,4
0,0
200
300
400
500
600
700

Figure S8.

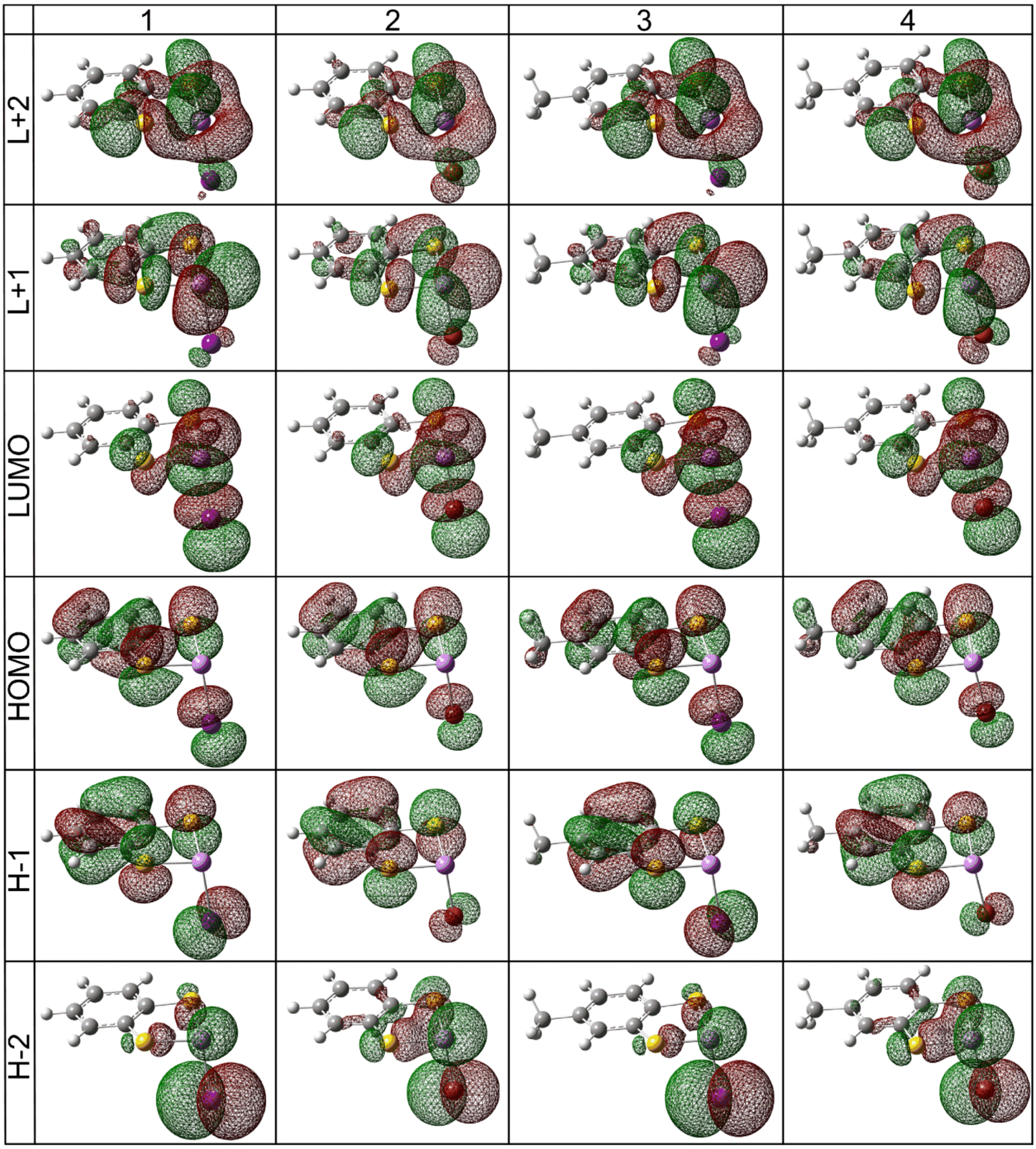

Tables S5. Calculated transitions for $AsI(PhS_2)$ (**1**).

Wavelength [nm]	Oscillator strength	Main components	%
381.49	0.1485	HOMO → LUMO	94.17
		HOMO → LUMO+2	2.63
355.05	0.0055	HOMO-1 → LUMO	94.17
		HOMO-3 → LUMO	3.29
342.02	0.0086	HOMO-2 → LUMO	98.70
332.22	0.0031	HOMO → LUMO+1	95.58
316.86	0.0009	HOMO-3 → LUMO	93.73
		HOMO-1 → LUMO	3.37
306.61	0.0194	HOMO → LUMO+2	86.24
		HOMO-1 → LUMO+1	10.81
291.00	0.0729	HOMO-1 → LUMO+1	84.80%
		HOMO → LUMO+2	9.28%
		HOMO → LUMO+3	2.36%
276.42	0.0012	HOMO-2 → LUMO+1	90.80%
		HOMO-1 → LUMO+2	7.36%
273.45	0.0066	HOMO-1 → LUMO+2	88.07%
		HOMO-2 → LUMO+1	6.94%
265.42	0.0335	HOMO-4 → LUMO	51.41%
		HOMO-3 → LUMO+1	27.52%
		HOMO → LUMO+3	10.94%
		HOMO-2 → LUMO+2	4.71%
		HOMO-1 → LUMO+4	3.46%
261.42	0.0305	HOMO → LUMO+3	58.10%
		HOMO-4 → LUMO	18.07%

		HOMO-1 → LUMO+4	9.36%
		HOMO-2 → LUMO+2	8.62%
255.86	0.0169	HOMO-3 → LUMO+1	46.52%
		HOMO-2 → LUMO+2	37.29%
		HOMO → LUMO+3	9.87%
		HOMO-1 → LUMO+4	2.58%
247.28	0.0049	HOMO-5 → LUMO	67.00%
		HOMO → LUMO+4	23.69%
		HOMO-1 → LUMO+3	4.25%
		HOMO-3 → LUMO+2	2.31%
245.36	0.0218	HOMO-5 → LUMO	67.00%
		HOMO → LUMO+4	50.69%
		HOMO-3 → LUMO+2	10.36%
		HOMO-1 → LUMO+3	3.16%
240.87	0.2587	HOMO-2 → LUMO+2	43.86%
		HOMO-4 → LUMO	19.85%
		HOMO-3 → LUMO+1	19.57%
		HOMO-1 → LUMO+4	4.82%
		HOMO-8 → LUMO	3.85%
		HOMO-10 → LUMO	2.44%
238.46	0.0042	HOMO-3 → LUMO+2	84.18%
		HOMO → LUMO+4	7.46%
		HOMO-1 → LUMO+3	3.65%
232.53	0.0076	HOMO-4 → LUMO+1	96.12%
226.59	0.0442	HOMO-1 → LUMO+3	83.09%
		HOMO →	7.02%

		LUMO+4	
		HOMO-5 → LUMO+3	2.22%
224.78	0.3672	HOMO-1 → LUMO+4	61.36%
		HOMO → LUMO+3	11.92%
		HOMO-4 → LUMO+2	10.96%
		HOMO-6 → LUMO	3.73%
		HOMO-4 → LUMO+3	3.39%
219.55	0.0038	HOMO-2 → LUMO+3	98.58%
218.21	0.0151	HOMO-4 → LUMO+2	59.14%
		HOMO-6 → LUMO	20.85%
		HOMO-5 → LUMO+1	6.01%
		HOMO → LUMO+5	3.52%
		HOMO → LUMO+6	3.34%
		HOMO-1 → LUMO+4	3.00%
215.00	0.0011	HOMO-2 → LUMO+4	97.11%
214.82	0.0316	HOMO → LUMO+5	87.84%
		HOMO-5 → LUMO+1	5.74%
		HOMO-4 → LUMO+2	2.50%
213.31	0.0644	HOMO-5 → LUMO+1	64.25%
		HOMO-4 → LUMO+2	14.49%
		HOMO-6 → LUMO	5.54%
		HOMO → LUMO+5	3.53%
		HOMO-1 → LUMO+4	3.18%
		HOMO-4 → LUMO+3	2.51%
211.69	0.0160	HOMO-7 → LUMO	75.11%
		HOMO-5 → LUMO+2	18.97%

209.96	0.0448	HOMO-6 → LUMO	55.51%
		HOMO-5 → LUMO+1	17.60%
		HOMO-4 → LUMO+2	7.69%
		HOMO → LUMO+6	5.28%
		HOMO-3 → LUMO+4	3.55%
		HOMO-1 → LUMO+4	3.27%
209.55	0.0221	HOMO → LUMO+6	82.25%
		HOMO-6 → LUMO	6.24%
		HOMO → LUMO+8	2.86%
		HOMO-3 → LUMO+4	2.14%
207.46	0.0132	HOMO-3 → LUMO+3	94.26%
205.05	0.0167	HOMO-3 → LUMO+4	82.74%
		HOMO-4 → LUMO+3	8.17%
		HOMO-6 → LUMO	3.15%
201.14	0.0156	HOMO-1 → LUMO+5	42.77%
		HOMO-5 → LUMO+2	30.69%
		HOMO-7 → LUMO	9.76%
		HOMO → LUMO+8	3.58%
		HOMO → LUMO+7	2.91%
		HOMO-6 → LUMO+1	2.12%

Tables S6. Calculated transitions for AsBr(PhS_2) (**2**).

**Wavelength [nm]**	**Oscillator strength**	**Main components**	**%**
345.16	0.0686	HOMO → LUMO	88.42
		HOMO → LUMO+2	9.63
320.20	0.0006	HOMO → LUMO+1	89.84
		HOMO-1 → LUMO	8.54
310.46	0.0060	HOMO-1 → LUMO	85.77
		HOMO →	8.67

		LUMO+1	
		HOMO-1 → LUMO+2	2.88
301.80	0.0640	HOMO → LUMO+2	84.65
		HOMO → LUMO	8.29
		HOMO-1 → LUMO+1	4.40
281.26	0.0407	HOMO-1 → LUMO+1	90.74
		HOMO → LUMO+2	3.57
		HOMO → LUMO+3	3.23
272.15	0.0262	HOMO-2 → LUMO	88.80
		HOMO-1 → LUMO+2	7.69
271.59	0.0070	HOMO-1 → LUMO+2	84.02
		HOMO-2 → LUMO+	8.01
		HOMO-1 → LUMO	2.48
		HOMO → LUMO+4	2.19
261.55	0.0238	HOMO → LUMO+3	74.28
		HOMO-1 → LUMO+4	19.62
		HOMO-1 → LUMO+1	2.49
251.53	0.0008	HOMO-3 → LUMO	90.62
		HOMO-2 → LUMO+1	5.45
248.97	0.0085	HOMO → LUMO+4	56.12
		HOMO-2 → LUMO+1	33.13
		HOMO-1 → LUMO+3	4.53
		HOMO-3 → LUMO	3.08
239.03	0.0482	HOMO-2 → LUMO+1	57.58
		HOMO → LUMO+4	24.42
		HOMO-1 → LUMO+3	7.53
		HOMO-3 → LUMO	2.10
234.48	0.0334	HOMO-2 → LUMO+2	66.20
		HOMO-4 → LUMO	29.74

228.15	0.0938	HOMO-4 → LUMO	38.16
		HOMO-3 → LUMO+1	31.43
		HOMO-2 → LUMO+2	12.42
		HOMO-1 → LUMO+4	9.42
		HOMO → LUMO+3	3.44
225.64	0.0404	HOMO-5 → LUMO	46.48
		HOMO-1 → LUMO+3	39.39
		HOMO → LUMO+4	4.83
		HOMO-3 → LUMO+1	2.03
223.84	0.2711	HOMO-5 → LUMO	29.41
		HOMO-1 → LUMO+4	23.19
		HOMO-1 → LUMO+3	18.51
		HOMO-3 → LUMO+1	11.81
		HOMO → LUMO+3	5.40
		HOMO → LUMO+4	2.07
223.44	0.2250	HOMO-1 → LUMO+4	32.27
		HOMO-1 → LUMO+3	21.81
		HOMO-5 → LUMO	16.06
		HOMO-3 → LUMO+1	12.92
		HOMO → LUMO+3	5.31
		HOMO-3 → LUMO+2	2.24
216.99	0.0129	HOMO → LUMO+5	87.11
		HOMO-3 → LUMO+1	3.80
215.37	0.0110	HOMO-3 → LUMO+2	89.09
213.58	0.1855	HOMO-3 → LUMO+1	31.25
		HOMO-4 → LUMO	21.61
		HOMO-2 → LUMO+2	13.35
		HOMO → LUMO+5	7.52

		HOMO-1 → LUMO+4	5.37
		HOMO-4 → LUMO+2	5.77
		HOMO → LUMO+3	2.79
		HOMO-10 → LUMO	2.52
		HOMO-5 → LUMO+1	2.01
211.94	0.0012	HOMO-4 → LUMO+1	96.71
209.41	0.0157	HOMO → LUMO+6	82.82
		HOMO-4 → LUMO+2	4.28
		HOMO → LUMO+8	3.99
205.97	0.0727	HOMO-5 → LUMO+1	80.73
		HOMO-4 → LUMO+2	3.46
		HOMO-6 → LUMO	2.39
		HOMO → LUMO+6	2.30
204.15	0.0233	HOMO-4 → LUMO+2	77.97
		HOMO-5 → LUMO+1	7.95
		HOMO → LUMO+6	4.38
203.54	0.0240	HOMO-2 → LUMO+3	93.99
		HOMO-5 → LUMO+1	2.13
202.34	0.0034	HOMO-1 → LUMO+5	47.90
		HOMO-5 → LUMO+2	27.17
		HOMO-6 → LUMO	9.28
		HOMO-7 → LUMO	4.49
		HOMO → LUMO+7	2.41
		HOMO-1 → LUMO+6	2.21
201.19	0.0028	HOMO-5 → LUMO+2	35.11
		HOMO-1 → LUMO+5	33.30
		HOMO-2 → LUMO+4	13.62

		HOMO-6 → LUMO	6.34
		HOMO-7 → LUMO	3.92
		HOMO → LUMO+7	2.27
200.82	0.0070	HOMO-2 → LUMO+4	81.82
		HOMO-1 → LUMO+5	7.59
		HOMO-5 → LUMO+2	2.71
		HOMO-7 → LUMO	2.07

Tables S7. Calculated transitions for AsI(MePhS_2) (**3**).

**Wavelength [nm]**	**Oscillator strength**	**Main components**	**%**
392.75	0.1447	HOMO → LUMO	94.89
		HOMO → LUMO+2	2.36
358.86	0.0082	HOMO-1 → LUMO	93.18
		HOMO-3 → LUMO	2.73
		HOMO → LUMO+1	2.07
342.56	0.0079	HOMO-2 → LUMO	98.65
338.15	0.0041	HOMO → LUMO+1	95.63
319.14	0.0009	HOMO-3 → LUMO	94.97
		HOMO-1 → LUMO	2.76
311.86	0.0236	HOMO → LUMO+2	89.82
		HOMO-1 → LUMO+1	6.83
293.73	0.0674	HOMO-1 → LUMO+1	88.63
		HOMO → LUMO+2	5.81
		HOMO → LUMO+3	2.25
276.55	0.0012	HOMO-1 → LUMO+2	62.67
		HOMO-2 → LUMO+1	34.66
274.97	0.0065	HOMO-2 → LUMO+1	62.64
		HOMO-1 → LUMO+2	32.63
270.23	0.0454	HOMO-4 → LUMO	71.14
		HOMO-3 → LUMO+1	16.82
		HOMO-2 → LUMO+2	6.88
263.63	0.0349	HOMO → LUMO+3	68.21
		HOMO-1 → LUMO+4	12.08
		HOMO-3 → LUMO+1	4.36

		HOMO-4 → LUMO	3.94
		HOMO-2 → LUMO+2	2.71
		HOMO → LUMO+4	2.50
256.17	0.0122	HOMO-3 → LUMO+1	53.29
		HOMO-2 → LUMO+2	35.34
		HOMO → LUMO+3	6.26
249.24	0.0090	HOMO-5 → LUMO	68.30
		HOMO → LUMO+4	21.28
		HOMO-1 → LUMO+3	3.70
246.83	0.0312	HOMO → LUMO+4	51.25
		HOMO-5 → LUMO	26.58
		HOMO-3 → LUMO+2	7.07
		HOMO-1 → LUMO+3	4.79
		HOMO → LUMO+3	2.21
241.77	0.2510	HOMO-2 → LUMO+2	48.25
		HOMO-3 → LUMO+1	20.30
		HOMO-4 → LUMO	14.40
		HOMO-1 → LUMO+4	3.32
		HOMO-8 → LUMO	2.74
239.18	0.0053	HOMO-3 → LUMO+2	88.98
		HOMO → LUMO+4	4.68
		HOMO-1 → LUMO+3	2.07
236.45	0.0072	HOMO-4 → LUMO+1	95.76
227.03	0.2733	HOMO-1 → LUMO+3	58.86
		HOMO-4 → LUMO+2	9.48
		HOMO →	8.33

		LUMO+4	
		HOMO-1 → LUMO+4	6.88
		HOMO-6 → LUMO	3.15
		HOMO → LUMO+3	2.70
224.66	0.1706	HOMO-1 → LUMO+4	47.39
		HOMO-1 → LUMO+3	20.77
		HOMO-4 → LUMO+2	14.74
		HOMO → LUMO+3	6.47
		HOMO-4 → LUMO+3	2.06
222.35	0.0292	HOMO-4 → LUMO+2	42.21
		HOMO-6 → LUMO	40.59
		HOMO-1 → LUMO+4	5.80
		HOMO-5 → LUMO+1	4.20
218.50	0.0389	HOMO → LUMO+5	95.61
217.82	0.0018	HOMO-2 → LUMO+3	97.00
216.58	0.0751	HOMO-6 → LUMO	36.37
		HOMO-4 → LUMO+2	26.68
		HOMO-1 → LUMO+4	11.88
		HOMO-5 → LUMO+1	8.34
		HOMO-4 → LUMO+3	2.71
		HOMO → LUMO+3	2.62
		HOMO-4 → LUMO	2.05
213.38	0.0422	HOMO-5 → LUMO+1	72.45
		HOMO-6 → LUMO	12.11
		HOMO-7 → LUMO	5.34
		HOMO-2 → LUMO+4	3.59

213.12	0.0018	HOMO-2 → LUMO+4	76.67
		HOMO-7 → LUMO	16.97
		HOMO-5 → LUMO+2	3.17
212.23	0.0227	HOMO-7 → LUMO	53.78
		HOMO-2 → LUMO+4	17.04
		HOMO-5 → LUMO+2	15.68
		HOMO-5 → LUMO+1	8.32
211.76	0.0306	HOMO → LUMO+6	82.57
		HOMO → LUMO+8	5.01
		HOMO → LUMO+7	4.12
207.13	0.0145	HOMO-3 → LUMO+3	85.79
		HOMO-3 → LUMO+4	5.95
203.54	0.0107	HOMO-3 → LUMO+4	78.46
		HOMO-4 → LUMO+3	7.07
		HOMO-3 → LUMO+3	6.53
202.66	0.0101	HOMO-1 → LUMO+5	65.40
		HOMO-5 → LUMO+2	9.36
		HOMO → LUMO+7	4.99
		HOMO → LUMO+8	4.40
		HOMO-7 → LUMO	3.81
		HOMO → LUMO+9	3.10
		HOMO-3 → LUMO+4	2.09

Tables S8. Calculated transitions for AsBr(MePhS$_2$) (**4**).

Wavelength [nm]	Oscillator strength	Main components	%
354.59	0.0676	HOMO → LUMO	89.21
		HOMO → LUMO+2	8.97
326.12	0.0009	HOMO-1 → LUMO	93.11
		HOMO → LUMO+1	5.16
314.95	0.0052	HOMO-1 → LUMO	87.02
		HOMO-1 → LUMO+2	5.15
		HOMO → LUMO+1	3.06
		HOMO → LUMO+2	2.13
308.52	0.0700	HOMO-1 → LUMO	84.73
		HOMO-1 → LUMO+1	7.80
		HOMO → LUMO	2.62
		HOMO → LUMO+2	2.36
283.96	0.0346	HOMO-1 → LUMO+1	91.15
		HOMO → LUMO+2	3.00
		HOMO → LUMO+3	2.20
274.81	0.0061	HOMO-1 → LUMO	89.98
		HOMO-1 → LUMO+2	2.60
		HOMO → LUMO+3	2.11
272.50	0.0284	HOMO-2 → LUMO	96.25
264.68	0.0213	HOMO-1 → LUMO+3	69.03
		HOMO → LUMO+4	15.25
		HOMO-1 → LUMO+1	5.99
		HOMO-1 → LUMO+4	3.20
		HOMO → LUMO+3	2.32
252.30	0.0009	HOMO-2 →	90.76

		LUMO+1	
		HOMO-4 → LUMO	2.83
		HOMO-3 → LUMO	2.52
250.39	0.0205	HOMO → LUMO+3	57.49
		HOMO → LUMO+4	23.91
		HOMO-2 → LUMO+1	8.22
		HOMO-1 → LUMO+3	6.72
239.29	0.0458	HOMO → LUMO+4	68.61
		HOMO-3 → LUMO	12.71
		HOMO-1 → LUMO+3	6.02
		HOMO → LUMO+3	2.95
		HOMO-2 → LUMO+1	2.27
237.95	0.0094	HOMO-4 → LUMO	57.52
		HOMO-2 → LUMO+2	36.55
230.28	0.1775	HOMO-4 → LUMO	40.71
		HOMO-3 → LUMO+1	22.28
		HOMO-2 → LUMO+2	16.83
		HOMO-1 → LUMO+3	5.75
		HOMO-1 → LUMO+4	4.62
		HOMO → LUMO+4	2.27
226.79	0.1671	HOMO-5 → LUMO	42.29
		HOMO → LUMO+4	36.7
		HOMO-3 → LUMO+1	8.06
		HOMO-1 → LUMO+3	3.9
225.26	0.1936	HOMO-5 → LUMO	45.54
		HOMO-3 → LUMO+1	20.91

		HOMO-1 → LUMO+3	17.26
		HOMO-1 → LUMO+4	3.74
		HOMO → LUMO+4	2.83
223.04	0.2100	HOMO-3 → LUMO+2	61.5
		HOMO-3 → LUMO+1	9.42
		HOMO-5 → LUMO	6.66
		HOMO-1 → LUMO+3	6.37
		HOMO → LUMO+3	4.01
		HOMO-1 → LUMO+4	2.26
221.11	0.0245	HOMO → LUMO+5	92.16
217.21	0.0392	HOMO-3 → LUMO+1	57.15
		HOMO-4 → LUMO	19.34
		HOMO-4 → LUMO+2	3.93
		HOMO-4 → LUMO+1	3.77
		HOMO-2 → LUMO+2	3.54
		HOMO-1 → LUMO+3	2.20
216.42	0.0165	HOMO-5 → LUMO	82.39
		HOMO-4 → LUMO+1	6.98
		HOMO-3 → LUMO+2	2.18
214.89	0.0892	HOMO-3 → LUMO+1	30.46
		HOMO-2 → LUMO+2	18.55
		HOMO-4 → LUMO+2	14.20
		HOMO-3 → LUMO+2	8.83
		HOMO-4 → LUMO+1	8.30
		HOMO-4 → LUMO	4.60
		HOMO-1 →	2.58

		LUMO+4	
211.67	0.0189	HOMO → LUMO+7	60.82
		HOMO → LUMO+6	8.49
		HOMO → LUMO+8	8.48
		HOMO-1 → LUMO+4	6.59
		HOMO-4 → LUMO+2	2.81
208.92	0.0605	HOMO-6 → LUMO	64.21
		HOMO-2 → LUMO+2	12.59
		HOMO-4 → LUMO+2	3.48
		HOMO-3 → LUMO+1	3.26
		HOMO-4 → LUMO	2.57
		HOMO → LUMO+7	2.52
		HOMO → LUMO+6	2.34
206.16	0.0660	HOMO-6 → LUMO	89.20
		HOMO-5 → LUMO+1	3.56
204.06	0.0046	HOMO-2 → LUMO+3	75.69
		HOMO → LUMO+7	5.70
		HOMO → LUMO+8	4.95
		HOMO-1 → LUMO+5	3.21
		HOMO-5 → LUMO+2	2.91
203.29	0.0158	HOMO-1 → LUMO+5	89.38
		HOMO-5 → LUMO+2	3.23
		HOMO-2 → LUMO+3	2.25
202.30	0.0012	HOMO-1 → LUMO+5	57.53
		HOMO-5 → LUMO+2	19.25
		HOMO → LUMO+8	5.29

		HOMO → LUMO+10	3.02
		HOMO-6 → LUMO	2.82
		HOMO-7 → LUMO	2.52
200.95	0.0053	HOMO → LUMO+6	75.02
		HOMO → LUMO+7	16.03
		HOMO → LUMO+8	2.31

Figure S9 a).

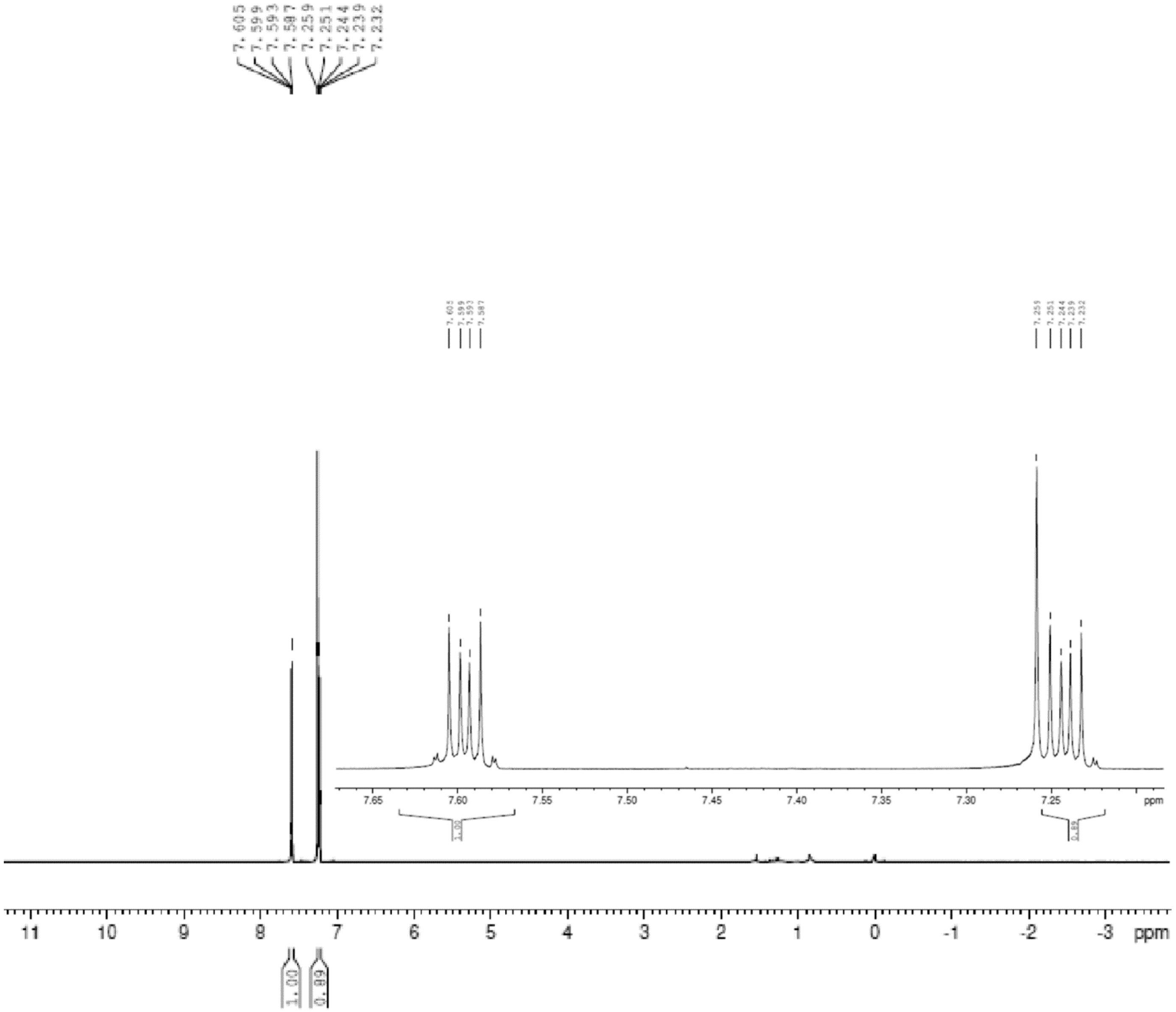

7.605
7.599
7.593
7.587
7.259
7.251
7.244
7.239
7.232
7.65
7.60
7.55
7.50
7.45
7.40
7.35
7.30
7.25
ppm
11
10
9
8
7
6
5
4
3
2
1
0
-1
-2
-3
ppm
1.00
0.89

Figure S9 b).

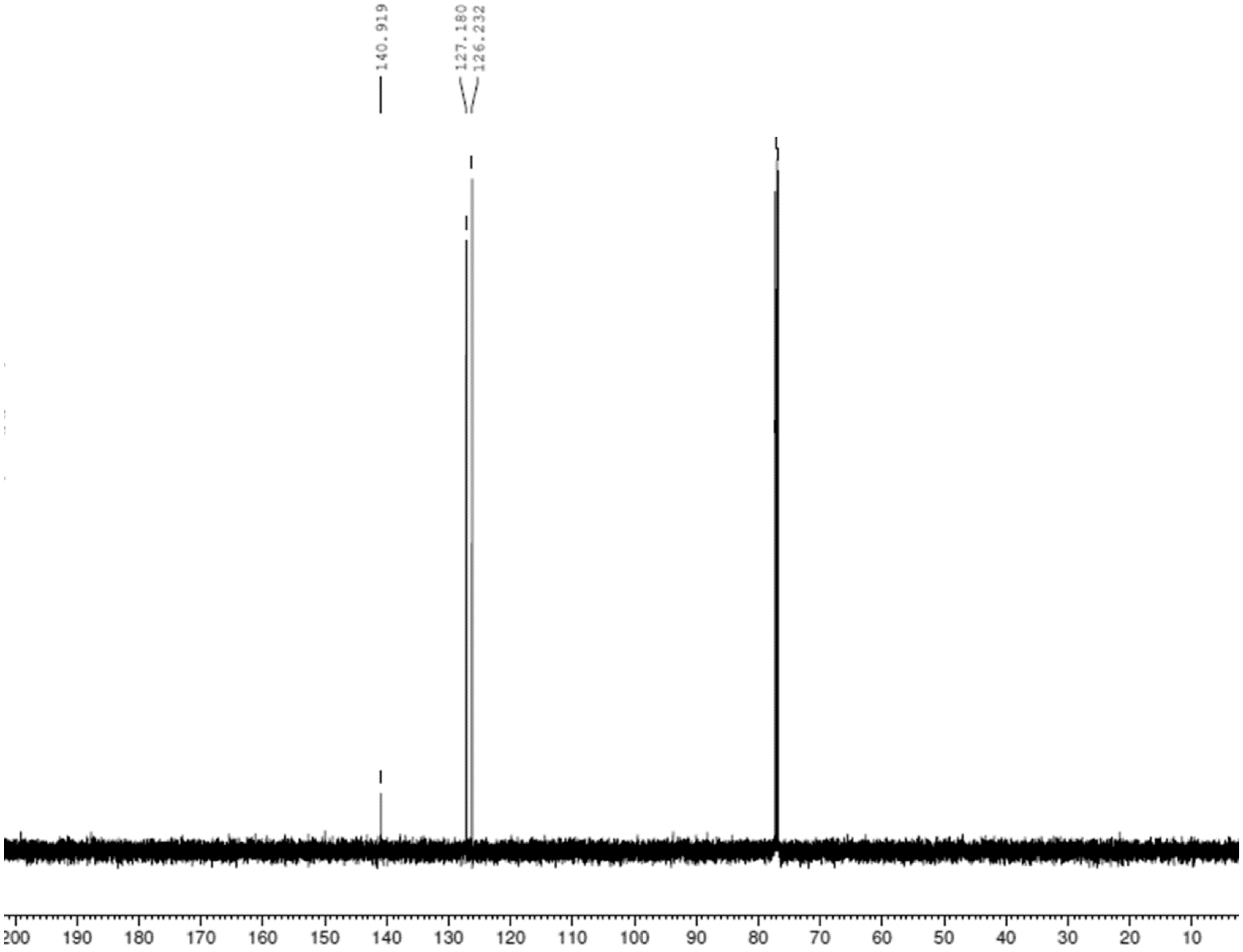
140.919
127.180
126.232
200
190
180
170
160
150
140
130
120
110
100
90
80
70
60
50
40
30
20
10

Figure S10 a).

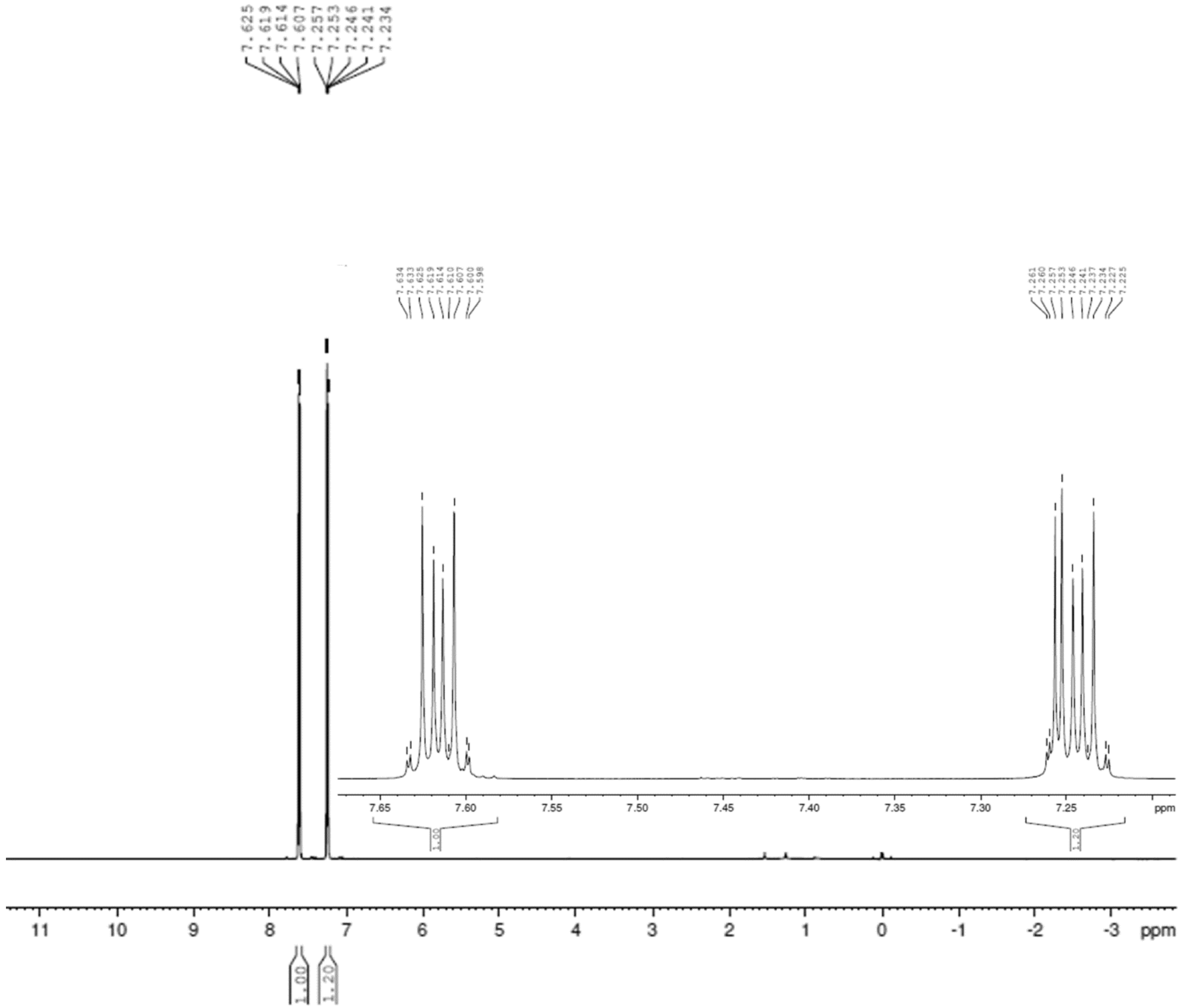

Figure S10 b).

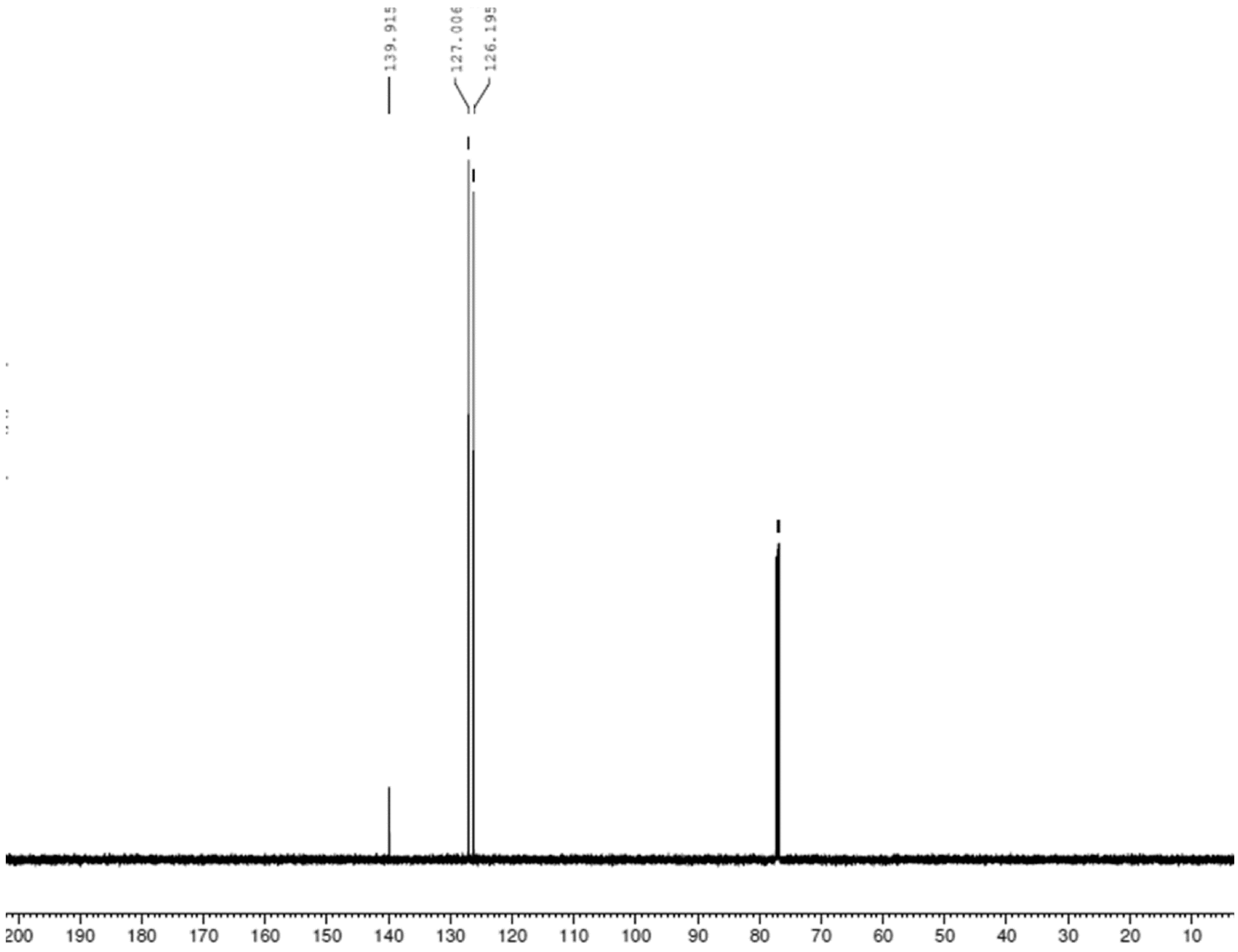

139.915
127.006
126.195
200
190
180
170
160
150
140
130
120
110
100
90
80
70
60
50
40
30
20
10

Figure S11 a).

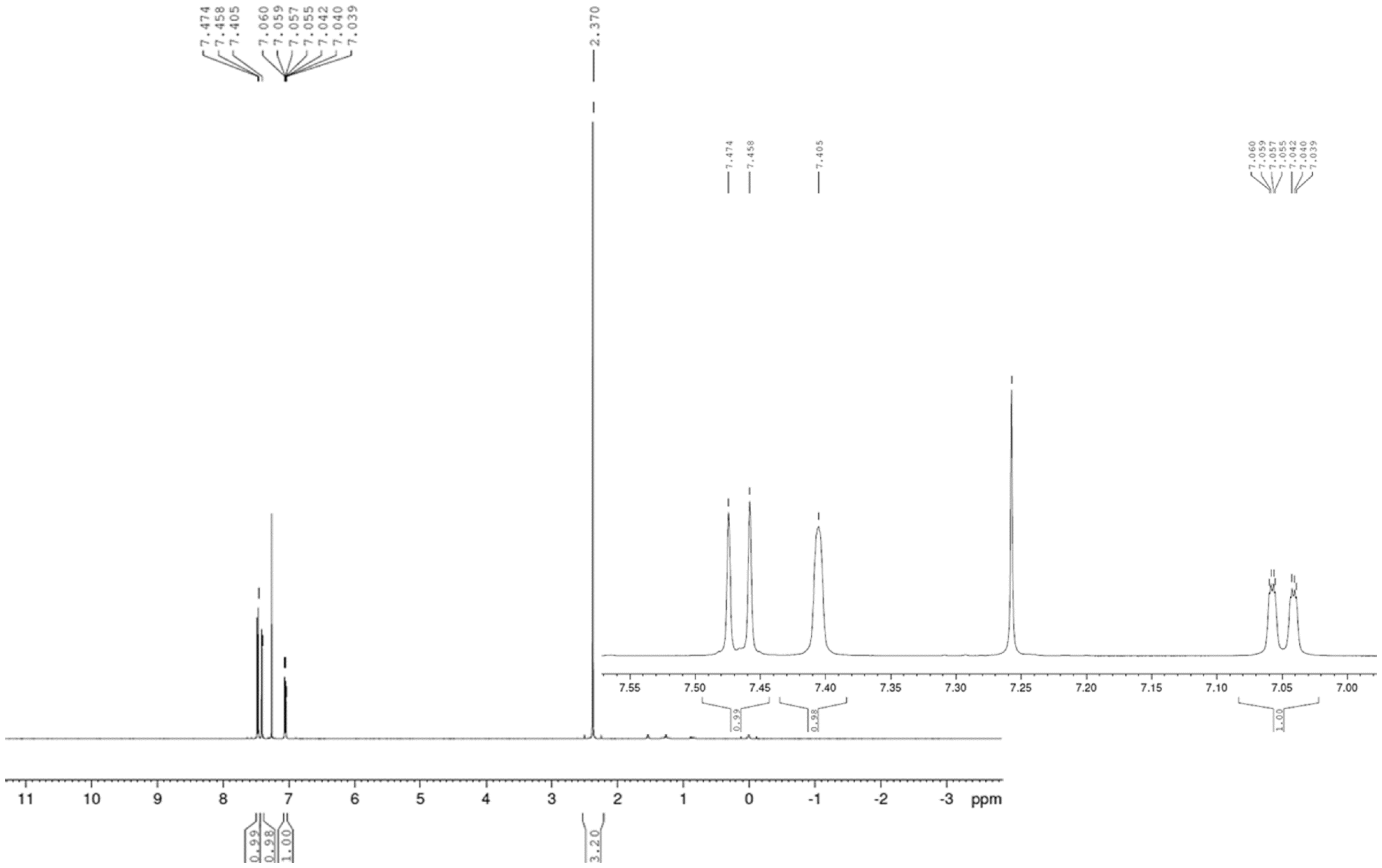
7.474
7.458
7.405
7.060
7.059
7.057
7.055
7.042
7.040
7.039
2.370
11 10 9 8 7 6 5 4 3 2 1 0 -1 -2 -3 ppm
0.99
0.98
1.00
3.20
7.55 7.50 7.45 7.40 7.35 7.30 7.25 7.20 7.15 7.10 7.05 7.00

Figure S11 b).

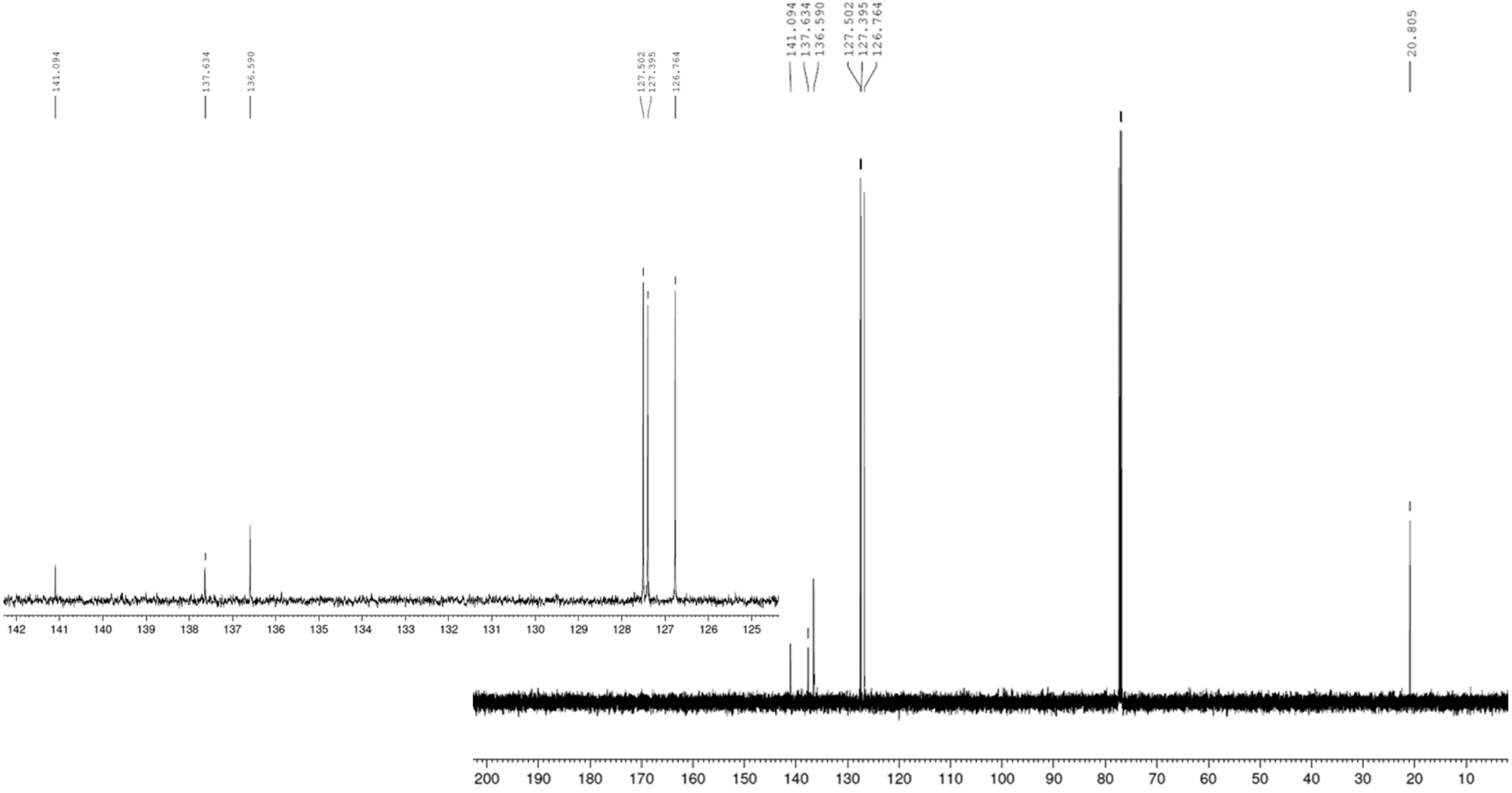

141.094
137.634
136.590
127.502
127.395
126.764
20.805
142
141
140
139
138
137
136
135
134
133
132
131
130
129
128
127
126
125
200
190
180
170
160
150
140
130
120
110
100
90
80
70
60
50
40
30
20
10

Figure S12 a).

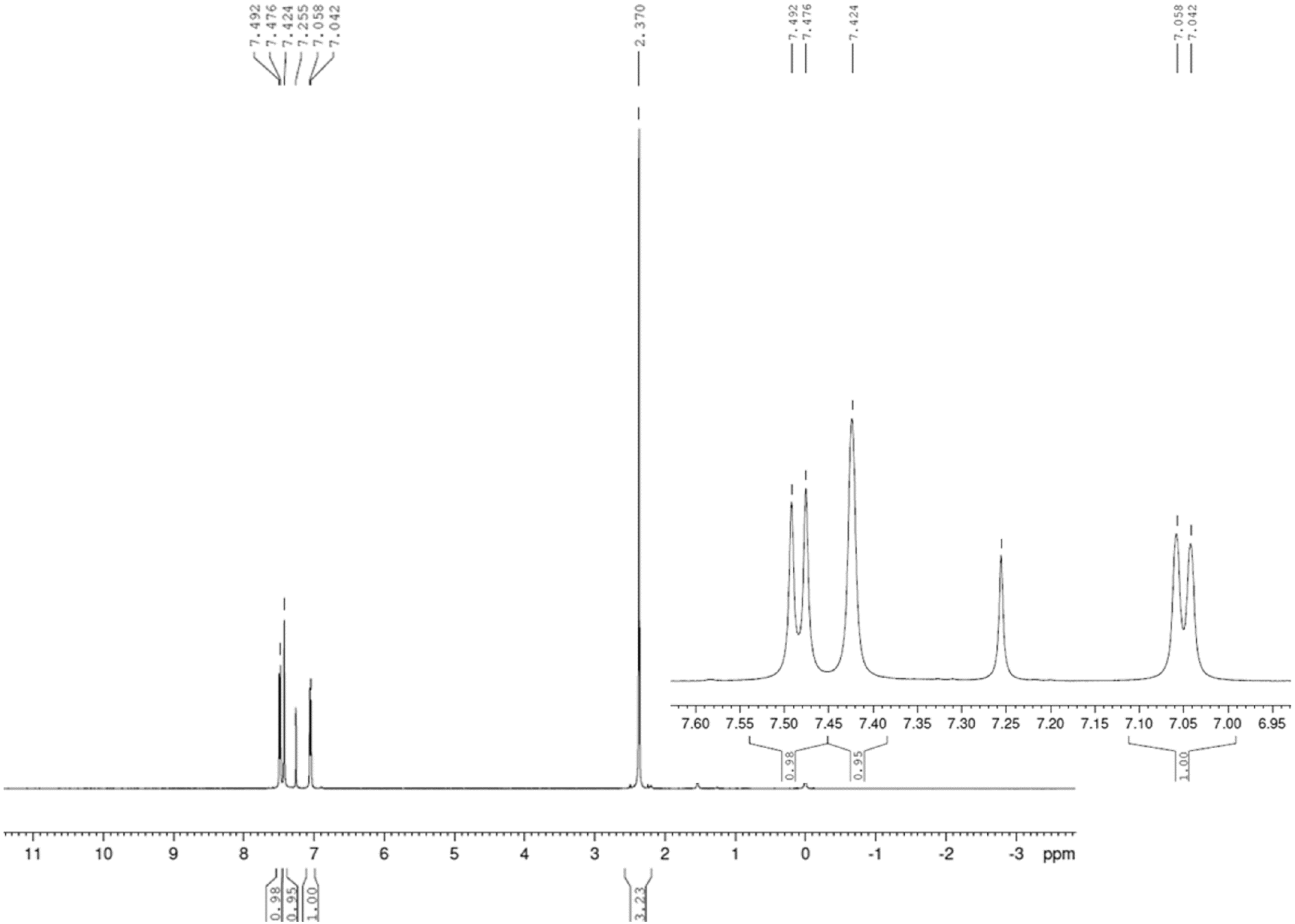

Figure S12 b).

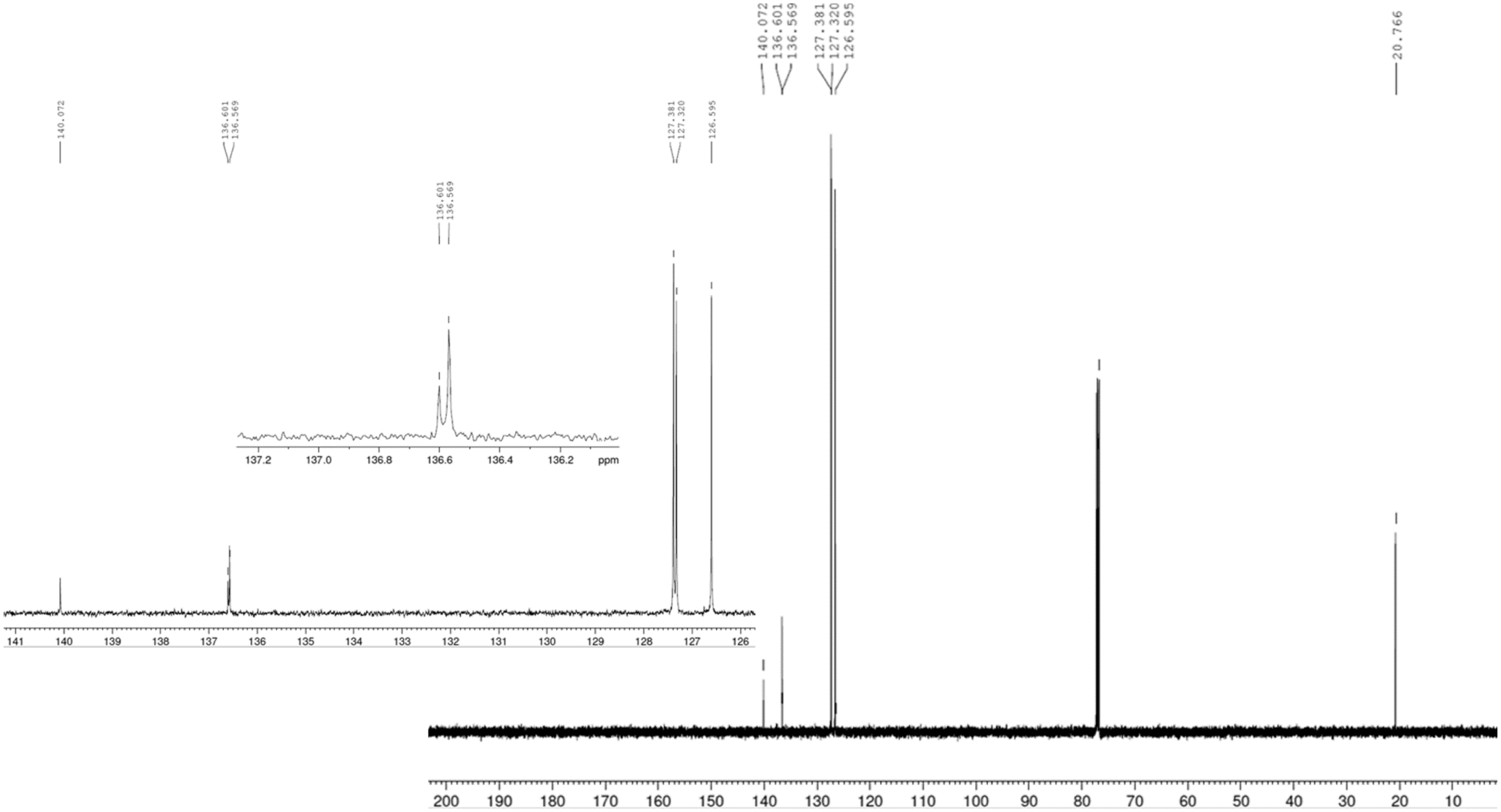
140.072
136.601
136.569
127.381
127.320
126.595
20.766
137.2
137.0
136.8
136.6
136.4
136.2
ppm
141
140
139
138
137
136
135
134
133
132
131
130
129
128
127
126
200
190
180
170
160
150
140
130
120
110
100
90
80
70
60
50
40
30
20
10
